# Discovering Potential RNA Dependent RNA Polymerase Inhibitors as Prospective Drugs Against COVID-19: An in silico Approach

**DOI:** 10.3389/fphar.2021.634047

**Published:** 2021-02-26

**Authors:** Satabdi Saha, Rajat Nandi, Poonam Vishwakarma, Amresh Prakash, Diwakar Kumar

**Affiliations:** ^1^ Department of Microbiology, Assam University, Silchar, India; ^2^ School of Computational and Integrative Sciences, Jawaharlal Nehru University, New Delhi, India; ^3^ Amity Institute of Integrative Sciences and Health, Amity University Haryana, Gurgaon, India

**Keywords:** COVID-19, RdRp, plant product, inhibitors, admet, free energy

## Abstract

COVID-19, caused by Severe Acute Respiratory Syndrome Corona Virus 2, is declared a Global Pandemic by WHO in early 2020. In the present situation, though more than 180 vaccine candidates with some already approved for emergency use, are currently in development against SARS-CoV-2, their safety and efficacy data is still in a very preliminary stage to recognize them as a new treatment, which demands an utmost emergency for the development of an alternative anti-COVID-19 drug *sine qua non* for a COVID-19 free world. Since RNA-dependent RNA polymerase (RdRp) is an essential protein involved in replicating the virus, it can be held as a potential drug target. We were keen to explore the plant-based product against RdRp and analyze its inhibitory potential to treat COVID-19. A unique collection of 248 plant compounds were selected based on their antiviral activity published in previous literature and were subjected to molecular docking analysis against the catalytic sub-unit of RdRp. The docking study was followed by a pharmacokinetics analysis and molecular dynamics simulation study of the selected best-docked compounds. Tellimagrandin I, SaikosaponinB2, Hesperidin and (-)-Epigallocatechin Gallate were the most prominent ones that showed strong binding affinity toward RdRp. All the compounds mentioned showed satisfactory pharmacokinetics properties and remained stabilized at their respective binding sites during the Molecular dynamics simulation. Additionally, we calculated the free-binding energy/the binding properties of RdRp-ligand complexes with the connection of MM/GBSA. Interestingly, we observe that SaikosaponinB2 gives the best binding affinity (∆G_binding_ = −42.43 kcal/mol) in the MM/GBSA assay. Whereas, least activity is observed for Hesperidin (∆G_binding_ = −22.72 kcal/mol). Overall our study unveiled the feasibility of the SaikosaponinB2 to serve as potential molecules for developing an effective therapy against COVID-19 by inhibiting one of its most crucial replication proteins, RdRp.

## Introduction

The widespread impact of COVID-19 has undoubtedly attained singular importance in the mind of international consciousness. The situation precipitated by the COVID-19 pandemic was certainly extraordinary in its rampant spread and impact across all walks of life. WHO had declared the Wuhan borne COVID-19 virus as a public health emergency of International Concern (PHEIC) (Ibrahim et al., 2020), and later, it was recognized as 2019-nCoV/SARS-CoV-2 (Zhou et al., 2020). It is a painful reflection of the rough times we live in and equally a reminder of how important it is for us to take precise, mature, and proactive action for an effective solution or therapy. Even though more than 180 vaccine candidates with some already approved for emergency use, are currently in development against the COVID-19 worldwide (Lythgoe and Middleton, 2020), the search for specific and effective small-molecule drugs for the treatment of COVID-19 would additionally provide another treatment strategy.

SARS-CoV-2 is an RNA virus belonging to the subgenus *Sarbecovirus* (beta-CoV lineage B). This strain has been reported to vary from the other beta-coronavirus, including the MERS-CoV and SARS I virus (Walls et al., 2020). SARS-CoV-2 codes for around 16 non-structural proteins (nsp), including RNA-dependent RNA polymerase (RdRp). RdRps share multiple sequence motifs and tertiary structures with all RNA viruses, making it one of the most lucrative targets for developing potential inhibitors. RdRps play a significant role in facilitating viral gene transcription and replication related to other viral and host factors (Gorbalenya et al., 2002). The RdRp is mainly composed of palm, thumb and finger domains, which resembles the typical right-hand RNA polymerase shape. Among the seven RdRp catalytic motifs, five (A–E) are present within the most conserved palm domain, while the other two (F and G) are within the finger domains (Gorbalenya et al., 2002; Venkataraman et al., 2018; Gao et al., 2020). The catalytic site of RDRp is conserved among different organisms and has two successive, surface-exposed aspartate residues projecting out from a beta-turn motif (Doublie and Ellenberger, 1998; Elfiky and Ismail, 2018; Elfiky and Azzam, 2020).

In this study, the SARS-CoV-2 RdRp homology model was built; it was subjected to Molecular docking, molecular dynamic simulation (MDS), MD Trajectory analysis, and MM-GBSA analysis. A structure-based virtual screening was employed in search of promising compounds as RdRp inhibitors from the PubChem database (https://pubchem.ncbi.nlm.nih.gov/). Two hundred forty-eight plant compounds comprised of flavonoids, alkaloids, lactones, and terpenes with antiviral activity against single-stranded RNA viruses were selected (Supplementary Table S1). Additionally, two nucleoside analogs, favipiravir, and remdesivir which, were recently approved for emergency COVID-19 treatment, have been taken as control. The molecular docking and binding affinity estimation process was used to screen all the natural compounds, including the controls, to compare its results with the hit molecules. The selected compounds were further examined through pharmacokinetics analysis.

The RdRp protein with its hit-molecules and inhibitors was subjected to a more in-depth analysis to extract its bio-molecules’ flexible nature, protein conformational changes, protein-ligand interactions, and structural perturbation, atomic detailing in context to time were thoroughly studied. These studies were carefully accomplished through an efficient and well-established computational method, namely MD simulation (Luthra et al., 2009; Mishra et al., 2018; Majewski et al., 2019; Wang et al., 2020). The further implication of MM-GBSA helped us to evaluate the various aspects of molecular interactions, such as the free binding energy estimation, effect of solvation, and thermodynamic integration (Wang et al., 2019; Kumar et al., 2020).

An extensive in-silico evaluation consisting of molecular docking, pharmacokinetics evaluation, MD simulation, and MM-GBSA were used to explore RdRp-hit molecules interactions' various aspects, leading to the selection of potential lead molecules for the development of promising RdRp inhibitors.

## Materials and Methods

### Homology Modeling

The structure of the SARS-CoV-2 RdRp protein (PDB ID: 6m71.1 A) was downloaded from the swiss-model webserver (Waterhouse et al., 2018) in a PDB format, which was further validated using PROCHECK at the EBI server (Laskowski et al., 1993).

### Energy Minimization and Model Validation

Energy minimization was performed to obtain a highly stable protein structure using the YASARA Energy minimization server (Krieger et al., 2009) and further validated using PROCHECK (Laskowski et al., 1993). Further ProSa were used to check the authenticity and the structural quality of the SARS-CoV-2 RdRp protein (Wiederstein and Sippl, 2007).

### Binding Site Prediction

A literature survey was done to predict the protein’s binding site (Gorbalenya et al., 2002; Venkataraman et al., 2018; Gao et al., 2020), cross-verified using the CASTp webserver. CASTp 3.0 provides dependable, inclusive, and global topological identifications and dimensions of protein designating residues' identification in the binding site pocket and its volume, cavities, and channels (Tian et al., 2018).

### Ligand Selection and Ligand file Preparation

A library of 248 plant compounds was prepared, mainly consist of flavonoids, alkaloids, lactones and terpenes. Compounds were selected based on their antiviral activity against single-stranded RNA virus (Lin et al., 2014). Additionally, two nucleoside analogs, favipiravir, and remdesivir were taken as control. SMILES for the selected ligands were taken from the PubChem (https://pubchem.ncbi.nlm.nih.gov/) (Supplementary Table S1) and converted to pdbqt format using Open Babel software.

### Molecular Docking

Molecular docking is the most critical part of computational drug designing, ensuring binding the ligand molecule to the selected protein's binding pocket in the right conformation. In the present study, PyRx virtual screening software was used in docking studies. PyRx uses autodock four and autodock vina as docking programs (Trott and Olson, 2010). A grid box was prepared to have a dimension of 120.6361 Å × 115.6029 Å × 116.6400 Å and 26.0286 Å × 44.5394 Å × 43.1279 Å in the *X*, *Y* and *Z* axis, respectively. The grid box covers almost all the active site residues. Compounds having the lowest binding energy were selected for further study. The docked protein-ligand structures were visualized in PyMol software (Lill and Danielson, 2011), and hydrogen bond interactions were studies using LigPlot (Wallace et al., 1995).

### Drug Likeliness and Pharmacological Properties

The selected ligand molecules' drug likeliness properties were predicted based on the Lipinski rule (Lipinski, 2004) and Molsoft L.L.C.: Drug-Likeness (http://www.molsoft.com/mprop/) webserver. The pkCSM tool (http://biosig.unimelb.edu.au/pkcsm/prediction) was used to predict the ADMET properties of the selected ligands (Douglas et al., 2015).

### Molecular Dynamics (MD) Simulation

All-atoms MD simulation was performed using Amber16, selecting ff14SB force field and TIP3P water molecules (Case et al., 2005; Maier et al., 2015) for the coordinates of RdRP and the docked complexes with drug molecules, Tellimagrandin I, SaikosaponinB2, Hesperidin, and (-)-Epigallocatechin Gallate. A GAFFs force field is used to parameterize all selected ligands (Wang et al., 2006). A cubic simulation box was prepared to keep the protein at the center with an edge distance of 10 Å, and the explicit TIP3P water molecules were padding around the protein (Jorgensen et al., 1983). The counter-ions (Na + Cl^−^) were added to neutralize the simulation box. Particle Mesh Ewald (PME), a cut-off of 14 Å, was applied for the electrostatic interactions, and a cut-off of about 12 Å was used to manage the Vander Waals forces (Essmann et al., 1995). The SHAKE algorithm was applied to constrain H-bonds (Ryckaert et al., 1977). The prepared systems’ energy minimization was performed in three stages, each of 10,000 steps of steepest descent (SD) and conjugate gradient (CG) to relax the system. Further, each simulation system was gradually heated from 50 to 300 K in six steps, followed by 10,000 steps of SD and CG minimization. Under the NVT ensemble condition, each system is equilibrated for 1 ns? Finally, all five systems were submitted for the production run under NPT ensemble condition for 100 ns with a time step of 2 fs?

### MD Trajectory Analysis and MM-GBSA Assay

From the obtained MD trajectories, using the cpptraj tool available in Amber16, the structural order parameters (RMSD, *R*_g_, RMSF, and SASA) were computed to analyze the structural stability of RdRP and binding complexes with drug molecules, Tellimagrandin I (-)-Epigallocatechin Gallate, SaikosaponinB2, and Hesperidin, respectively. The binding free energy of protein-ligand complexes was estimated utilizing the Molecular Mechanics/Generalized Born Surface Area (MM/GBSA) method, taking the structural ensembles from the last 20 ns trajectory (Wang et al., 2019; Kumar et al., 2021). The binding free energy components can be represented according to the equations:

$${\text{ΔG}=\text{G}}_{\text{comp}}-\left({\text{G}}_{\text{rec}}{+\text{G}}_{\text{lig}}\right),$$ (1)

where ∆G represents the binding free energy of the receptor-ligand system, G_comp_ denotes the free energy of receptor complexed with ligand and, G_rec_ and G_lig_ define the individual free energy of receptor and ligand, respectively. The binding free energy of each of these was calculated using the MMPBSA. py script (Amber16). The bonded and non-bonded energy terms, electrostatic interactions, and van der Waals energies are defined by molecular mechanics energy (EMM). The polar solvation free energies $\left({\text{G}}_{\text{SOL}-\text{GB}}\right)$ and nonpolar solvation free energies $\left({\text{G}}_{\text{SURF}}\right)$ are calculated from the solvent-accessible surface area, and $\left({\text{G}}_{\text{SOL}}={\text{G}}_{\text{SOL}-\text{GO}}+{\text{G}}_{\text{SCRF}}\right)$ using the generalized Born approach.

## Results

### Homology Modeling, Energy Minimization and Model Validation

The degree of sequence similarity between the template and the query amino acid sequence dramatically determines the generated models' solidity (Azam et al., 2014). Here, the PDB ID- 6m71.1 A having a sequence similarity of 100%, was selected as a template. SWISS-MODEL web server computed a ligand and co-factor free model with an excellent GMQE (Global Model Quality Estimation) score of 0.86 and QMEAN Z-scores −1.52 (Waterhouse et al., 2018). The RdRp model structure was downloaded, and the Ramachandran plot was further used to validate the model via PROCHECK (Laskowski et al., 1993). The predicted model had 100% residues in favored, additionally allowed, and generously allowed regions (Supplementary Figure S1).

YASARA Energy minimization webserver was used to enhance the modeled structure's stereochemistry (Supplementary Figure S2). RdRp structure was further evaluated through ProSa. The z-score was −12.9 (Supplementary Figure S3A **),** and ProSa designated energy plots (Supplementary Figure S3B) showed an excellent structure.

### Binding Site Prediction

Binding site residues or binding pockets were anticipated through a literature survey (Gorbalenya et al., 2002; Gao et al., 2020; Venkataraman et al., 2018) and a CASTp web server. The superimposed residues were considered as binding site residues **(** Supplementary Figure S4A,S4B **).** This structurally conserved RdRp core plays a vital role in viral RdRp enzymatic function and shows an excellent drug target (Shu and Gong, 2016). The palm region ranging from 582–628 amino acids, was considered a binding site for further studies (Supplementary Figure S4A,S4B).

### Molecular Docking and Interaction Study

The Plant compounds, composed of 248 molecules, were docked against the SARS-CoV-2 RdRp protein using PyRx software. To some extent, all the molecules interacted with the binding site of the target protein. However, out of 248 molecules, four molecules, namely Tellimagrandin I, SaikosaponinB2, Hesperidin, and (-)-Epigallocatechin Gallate, showed the best docking score of −9.6 kcal/mol, −8.9 kcal/mol, −8.6 kcal/mol, and −8.1 kcal/mol, respectively (Table 1). Two nucleoside analogs, namely favipiravir, and remdesivir, were taken as a control and docked against the RdRp protein with the same grid dimension used for the target protein and showed −5.7 and −6.9 kcal/mol docking score, respectively (Table 1).

**TABLE 1:** Binding energy values (kcal/mol) and interactions of the ligand with the key residue of RNA dependent DNA polymerase of SARS-CoV-2 evaluated by PyRx docking.

Ligand molecules	Binding energy (Kcal/mol)	Key residues interactions
Tellimagrandin I	−9.6	Asp617
		Tyr618
		Cys621
		Asp622
		Arg623
SaikosaponinB2	−8.9	Lys620
		Arg623
Hesperidin	−8.6	Lys620
		Asp622
		Arg623
(-)-Epigallocatechin gallate	−8.1	Asp617
		Asp622
		Cys621
Remdesivir	−6.9	–
Favipiravir	−5.7	–

**FIGURE 1:**
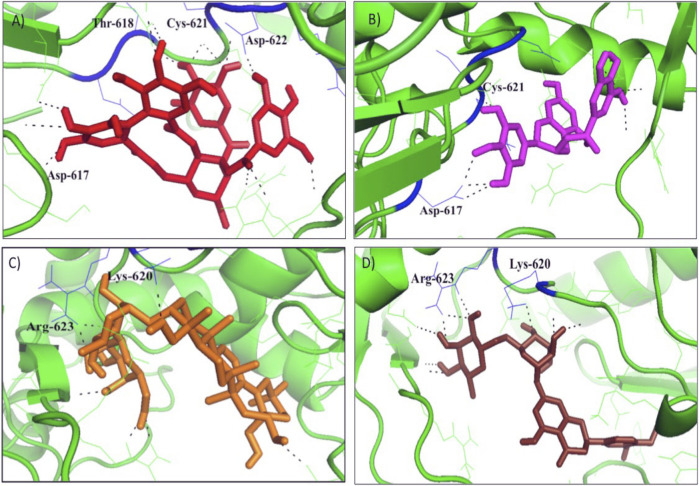
The docking results o**f (A)** Tellimagrandin I **(B)** (-)-Epigallocatechin Gallate **(C)** SaikosaponinB2, and **(D)** Hesperidin inside binding pocket of RNA dependent DNA polymerase (RdRp) of the SARS-CoV-2. Hydrogen bonded interactions are shown as black dotted lines.

**FIGURE 2:**
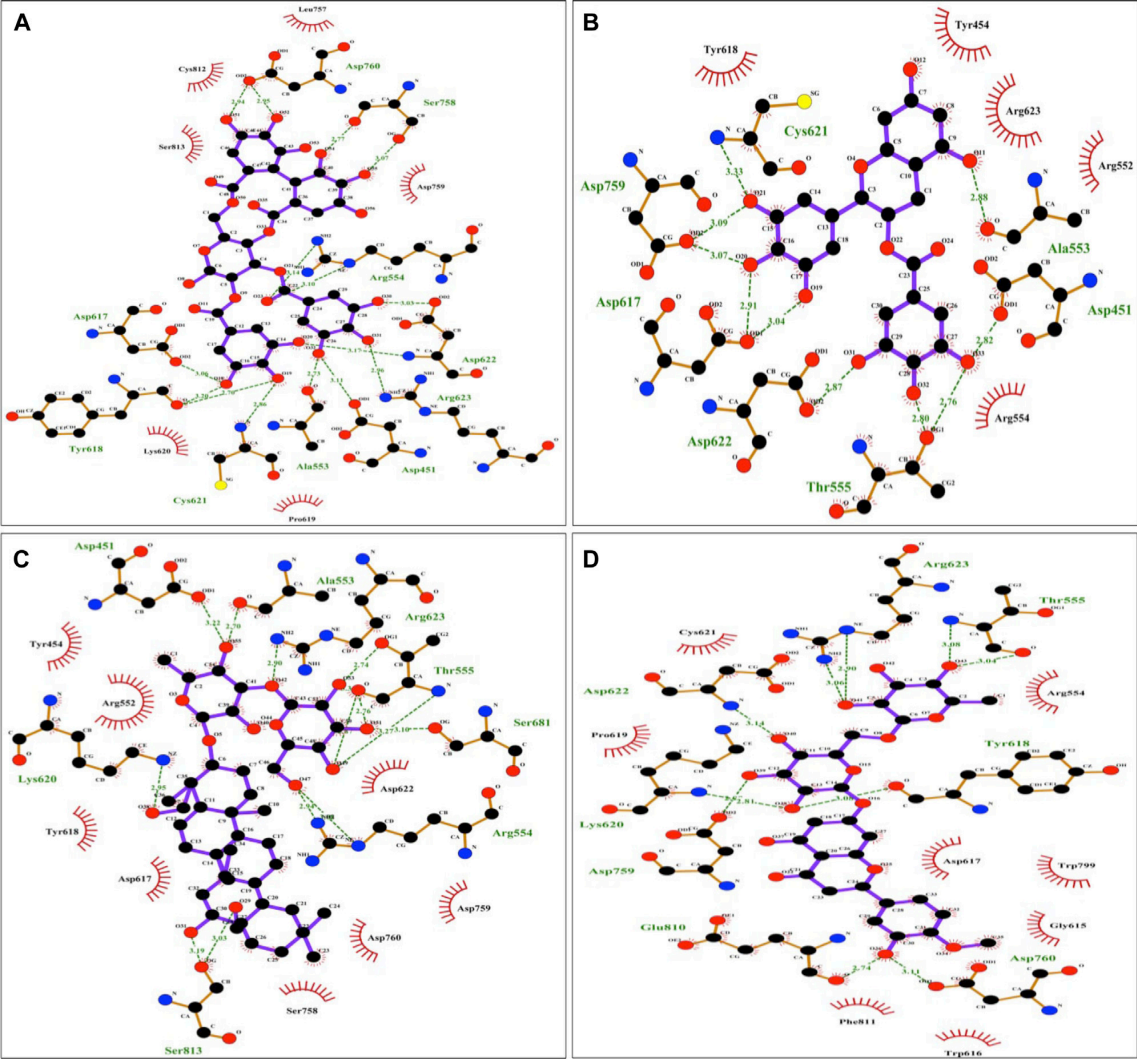
Diagrammatic sketch illustrating the interactions between **(A)** Tellimagrandin I **(B)** (-)-Epigallocatechin Gallate **(C)** SaikosaponinB2 **(D)** Hesperidin and RNA dependent DNA polymerase of the SARS-CoV-2 by Ligplot. Ligand is shown in purple and: green dashed lines indicate hydrogen bonds with distance in angstrom (Å), spoked red arcs indicate hydrophobic contacts, atoms are shown in black for carbon, blue for nitrogen, red represents oxygen, green represents fluorine, and yellow represents sulfur.

The protein-ligand interaction was visualized using PyMol software, and the best positions were selected (Figures 1A–D). LigPlot analysis revealed the hydrogen bond interaction between the targeted protein and ligand molecules (Figures 2A–D). It was noticeable that our four selected molecules formed strong H-bond interaction commonly with the amino acid residues- ASP 617, ASP 618, LYS 620, ASP 622, and ARG 623 exhibiting a great affinity between them.

The docking and interaction pattern of the top four ligand molecules shows that they are capable of binding with the catalytic palm region of the protein, thus inhibiting the activity of RdRp and blocking the viral replication and transcription. This study unlocked the door for the proposed molecules for further pharmacokinetic analysis.

### Pharmacokinetics Studies

Selected ligands were subjected to pharmacokinetics, including Lipinski rule 5, Drug likeness and ADMET analysis.

Result Obtained from the Lipinski rule of five are listed in Supplementary Table S2 (-)-Epigallocatechin Gallate satisfied all the Lipinski rule parameters. Whereas other molecules violate the Lipinski rule, previous studies suggest that saikosaponinB2 and tellimagrandin I have been known to have inhibitory activity against coronavirus 229 E and HCV, respectively (Cheng et al., 2006; Tamura et al., 2010). Again, the Hesperidin has antiviral activity against rotavirus (Bae et al., 2000). While (-)-Epigallocatechin Gallate was found effective against SARS-CoV-2 (Singh et al., 2020). However, all four molecules show favorable drug-likeness properties (Supplementary Table S2 and Supplementary Figures S5A–D).

pkCSM web tool was used to predict the ADMET properties of the selected molecules.

Absorption: Absorption is mainly calculated on account of water solubility, Caco2 permeability, human intestinal absorption, skin permeability, and whether the molecule is a P-glycoprotein substrate or inhibitor (Azam et al., 2014; Bhowmik et al., 2020; Sinha et al., 2020). The water solubility of the compound reflects at 25°C. All the selected molecules are moderately soluble in the water (Table 2). Caco2 permeability and human intestinal absorption determine the ultimate bioavailability; a drug having a value of more than 0.90 is considered readily permeable (Azam et al., 2014; Bhowmik et al., 2020; Sinha et al., 2020). All the ligands molecules show good permeability (Table 2).

**TABLE 2:** Absorption and distribution profile of the Tellimagrandin I, SaikosaponinB2, Hesperidin, and (-)-Epigallocatechin Gallate by pKCSM tool.

Compounds/Ligands	Water solubility log mol/L	Caco2 permeability log 10^–6^ cm/s	Human intestinal absorption (%)	P-glycoprotein substrate	P-glycoprotein I inhibitor	P-glycoprotein II inhibitor	VDss (log L/kg)	Fraction unbound (human)
Tellimagrandin I	−2.892	−1.605	61.586	Yes	Yes	Yes	0.112	0.353
SaikosaponinB2	−2.482	0.237	25.425	Yes	Yes	No	−0.35	0.343
Hesperidin	−3.014	0.505	31.481	Yes	No	No	0.996	0.101
(-)-Epigallocatechin gallate	−2.894	−1.521	47.395	Yes	No	Yes	0.806	0.215

The human intestine is the primary site where drugs usually get absorbed. Hydrophilic molecules are easily absorbed. A molecule with more than 30% absorbency is considered readily absorbed (Azam et al., 2014; Bhowmik et al., 2020; Sinha et al., 2020). Tellimagrandin I is found to be highly absorbed in the human intestine (Table 2). All the selected molecules are substrates for P-glycoprotein. Other than Hesperidin, all the molecules are either P-glycoprotein I or P-glycoprotein II inhibitors (Table 2). Thus, selected molecules could regulate P-glycoprotein's physiological function in the distribution of drugs.

Distribution: In the pKCSM tool, distribution is calculated in the following mentioned parameters - Human volume of distribution, human fraction unbound in plasma, blood-brain barrier, and central nervous system permeability. The volume of distribution is a theoretical volume that defines the drug’s overall dose, which needs to assort identically across to give a similar blood plasma concentration. The higher the VDss value, the more of a drug is distributed in tissue rather than plasma. More extensive tissue distribution is desirable for antibiotics and antivirals (Bhowmik et al., 2020; Sinha et al., 2020). VDss is considered low if the log VDss value is lower than −0.15, while the value higher than 0.45 is considered high (Bhowmik et al., 2020; Sinha et al., 2020). Among all the molecules**, hesperidin** shows the highest value, followed by **(-)Epigallocatechin Gallate, Tellimagrandin I, and SaikosaponinB2,** respectively (Table 2). Most plasma drugs will occur in symmetry in between unbound or bound states concerning serum proteins. The drug's effectiveness may be stirred by a limit to which it binds to the blood's proteins; as more can bind, it can transverse cellular membrane (Bhowmik et al., 2020; Sinha et al., 2020). Fraction unbound to human plasma should lie between 0.02 and 1.0 (Sinha et al., 2020). All the compounds show a good value (Table 2).

Metabolism: Metabolism of a drug depends upon the molecule to be a Cytochrome P450 substrate or inhibitor. All the selected molecules are **non-inhibitor** of any cytochrome enzyme, which indicates that they will be metabolized by the enzyme's action, suggesting that they will not be hampered through the body's biological transformation (Supplementary Table S3).

Excretion: Excretion is calculated with total clearance and whether the molecule is a renal OCT2 substrate. Organic cation transporter 2 (OCT2) is a renal uptake transporter that deposits and clears drugs from the kidney (Sinha et al., 2020). Only **Tellimagrandin I** acts as a substrate for Renal OCT2, while other drugs are removed via a different route. All the selected molecules show total clearance less than log(CLtot) 1 ml/min/kg (Table 3).

**TABLE 3:** Excretion and toxicity profile of the Tellimagrandin I, SaikosaponinB2, Hesperidin, and (-)-Epigallocatechin Gallate by pKCSM tool.

Compounds	Total clearance log ml/min/kg	Renal OCT2 substrate	AMES toxicity	Max. Tolerated dose (human)	Oral rat acute toxicity (LD50)	hERG inhibitor	Hepatotoxicity	Skin sensitization	Minnow toxicity
Tellimagrandin I	0.177	Yes	No	0.438	2.482	No	No	No	13.101
SaikosaponinB2	0.223	No	No	−2.178	2.959	No	No	No	1.934
Hesperidin	0.211	No	No	0.525	2.506	No	No	No	7.131
(-)-Epigallocatechin gallate	0.292	No	No	0.441	2.522	No	No	No	7.713

Toxicity: The AMES test showing a negative value indicates that it is non-mutagenic and non-carcinogenic. None of the selected ligand molecules shows positive AMES results (Table 3). The Maximum recommended tolerance dose (MRTD) provides an estimate of the toxic dose in humans. MRTD less than or equal to log 0.477 (mg/kg/day) is considered low (Sinha et al., 2020). All the compounds have low toxicity to humans (Table 3). **hERG** (human ether-a-go-go gene) is responsible for blocking potassium channels (Sinha et al., 2020). All the selected ligands are non-inhibitor of **hERG** and do not induce hepatotoxicity and non-skin sensitive (Table 3).

A molecule with a high oral rat acute toxicity (LD50) value is less lethal than the lower LD50 value (Bhowmik et al., 2020; Sinha et al., 2020). For a given compound, the LD50 is the amount that causes the death of 50% of the test animals (Bhowmik et al., 2020; Sinha et al., 2020). All the selected ligands showed high oral rat acute toxicity (LD50) value (Table 3). The lethal concentration values (LC50) represent a molecule’s concentration necessary to cause 50% of Fathead Minnows’ death. For a given compound, if the log LC50 < 0.5 mM (log LC50 < −0.3), then it is regarded as high acute toxic (Bhowmik et al., 2020; Sinha et al., 2020), all the compounds shows good score indicating that they are less toxic (Table 3).

### MD Simulation

**FIGURE 3:**
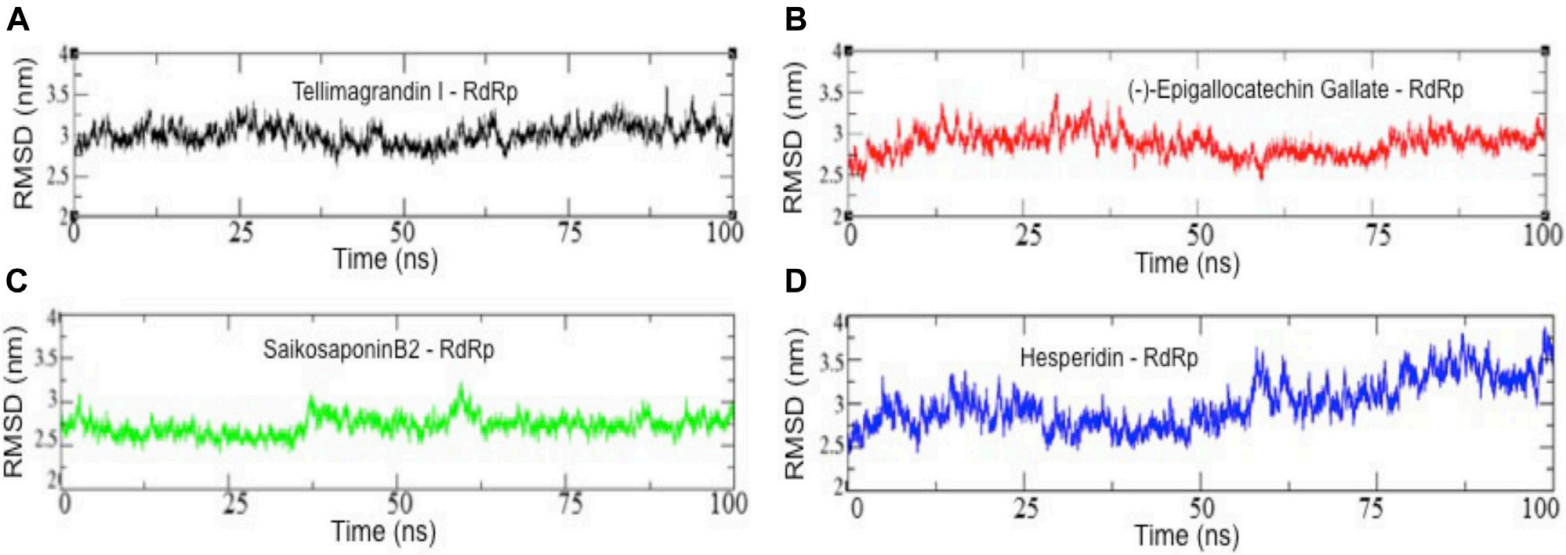
Backbone root mean square deviation (C^α^-RMSD) results of all four complexes. RMSD values of RdRp-Tellimagrandin I **(A)**, RdRp-(-)-Epigallocatechin Gallate **(B)**, RdRp-SaikosaponinB2 **(C)**, and RdRp-Hesperidin **(D)**. Molecular dynamic simulations were conducted for 100 ns?

To determine RdRp docked complexes’ conformation stability with drug molecules, Tellimagrandin I, SaikosaponinB2, Hesperidin, and (-)-Epigallocatechin Gallate, we computed the backbone root mean square deviation (C^α^-RMSD), as shown in Figure 3. The result shows that the RMSD trajectory of RdRp-Tellimagrandin I quickly attains equilibrium during 0–5 ns and remains steady with RMSD value 3.0 ± 0.2 Å at the end of simulation at 100 ns (Figure 3A). Similarly, the RMSD plot of the RdRp-SaikosaponinB2 complex shows a relatively stable structure during ∼0–37 ns with RMSD ∼2.57 Å. With slight drift, RMSD increase to ∼3.0 Å at ∼37 ns, which settles at ∼65 ns, and a stable equilibrium is continued for the remaining period of simulation (Figure 3C). However, RdRp-(-)-Epigallocatechin Gallate’s plot shows a gradual increase in RMSD during ∼0–35 ns, which slowly attains equilibrium at ∼50 ns and remains consistent around RMSD value ∼3.0 Å till 100 ns (Figure 3B). The conformational dynamics of RdRp- Hesperidin also shows an initial increase in RMSD up to ∼25 ns? The structure remains stable with RMSD ∼2.80 Å at ∼25–60 ns. Further increase in RMSD can be noticed due to several small drifts that settle at ∼85 ns, and the simulation ends with an increase in RMSD ∼3.50 Å (Figure 3D).

**FIGURE 4:**
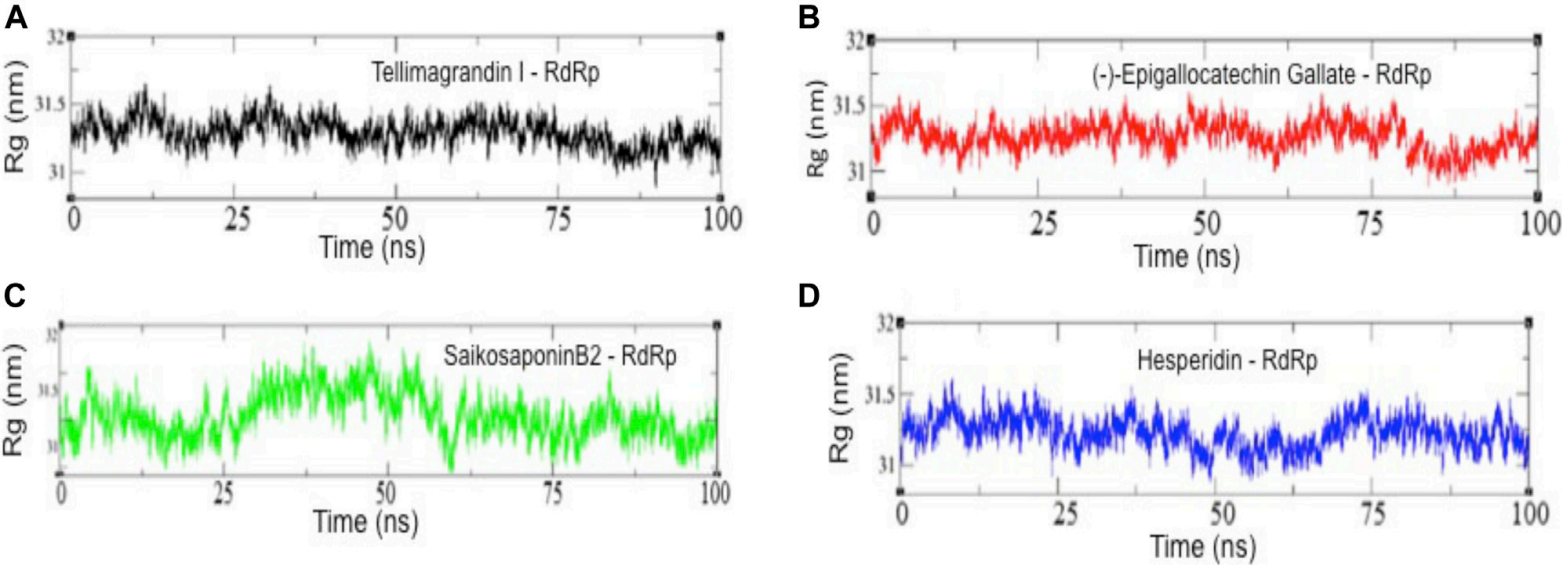
The radius of gyration (*R*_g_) results of all four complexes. Rg values of RdRp-Tellimagrandin I **(A)**, RdRp-(-)-Epigallocatechin Gallate **(B)**, RdRp-SaikosaponinB2 **(C)**, and RdRp-Hesperidin **(D)**. Molecular dynamic simulations were conducted for 100 ns?

To examine the structural compactness and integrity of RdRp-drug bound complexes, the radius of gyration (*R*_g_) is calculated for each system (Nygaard et al., 2017; Prakash et al., 2018a). Figure 4 shows that the structure of RdRp-Tellimagrandin I is stabilized around *R*_g_ value 31.25 Å, and we can see only the minor perturbations with small drifts of ∼0.20 Å at 0–25 ns, but it remains stable for the remaining period of simulation (Figure 4A). The *R*_g_ plot of RdRp-SaikosaponinB2 shows a slight drop down in trajectory during 0–25; after that, a drift of ∼0.70 Å can be seen at ∼25–30 ns, which settles gradually at ∼60 ns, and the structure remains stable for the period of 100 ns (Figure 4C). Whereas the conformational dynamics of RdRp-(-)-Epigallocatechin Gallate and RdRp-Hesperidin shows less perturbed structure throughout the simulation period with *R*_g_ value 31.25 Å (Figures 4B,D respectively).

**FIGURE 5:**
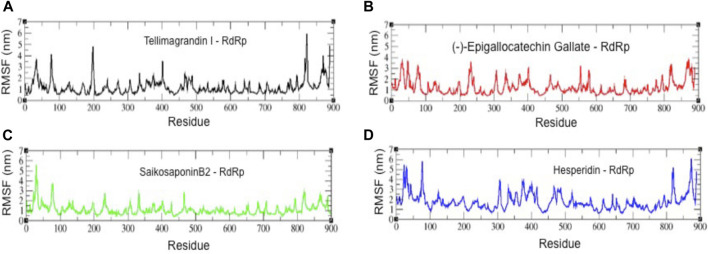
The root mean square fluctuation (RMSF) plots of all four complexes. RMSF plots of RdRp-Tellimagrandin I **(A)**, RdRp-(-)-Epigallocatechin Gallate **(B)**, RdRp-SaikosaponinB2 **(C)**, and RdRp-Hesperidin **(D)**. Molecular dynamic simulations were conducted for 100 ns?

We also analyzed the root mean square fluctuation (RMSF) plots of all four complexes (Figure 5). The residues belonging to stable secondary conformations (α-helix and β-sheet) show a lower degree of residual fluctuations, whereas the residues belonging to the terminal (N-and C-terminal) and loop regions have a high degree of fluctuations. The RMSF plots of RdRp-Tellimagrandin I, RdRp-(-)-Epigallocatechin Gallate, and RdRp-SaikosaponinB2 represent typical pattern profiles that the amino acid residues belonging to termini (N-and C-terminal) and loops have average atomic fluctuation >1.5 Å (Figures 5A–C respectively). In contrast, the conformational dynamics of stable secondary structure, α-helices, and β-sheets remain stable during the simulation, providing elegance evidence of the stable molecular interactions with ligands. However, the plot of RdRp-Hesperidin shows that along with the regions belonging to loops, the residues 300–400 also having comparatively higher average fluctuations >2.0 Å (Figure 5D). This result indicates the loosely bounded conformation of Hesperidin with RdRp.

**FIGURE 6:**
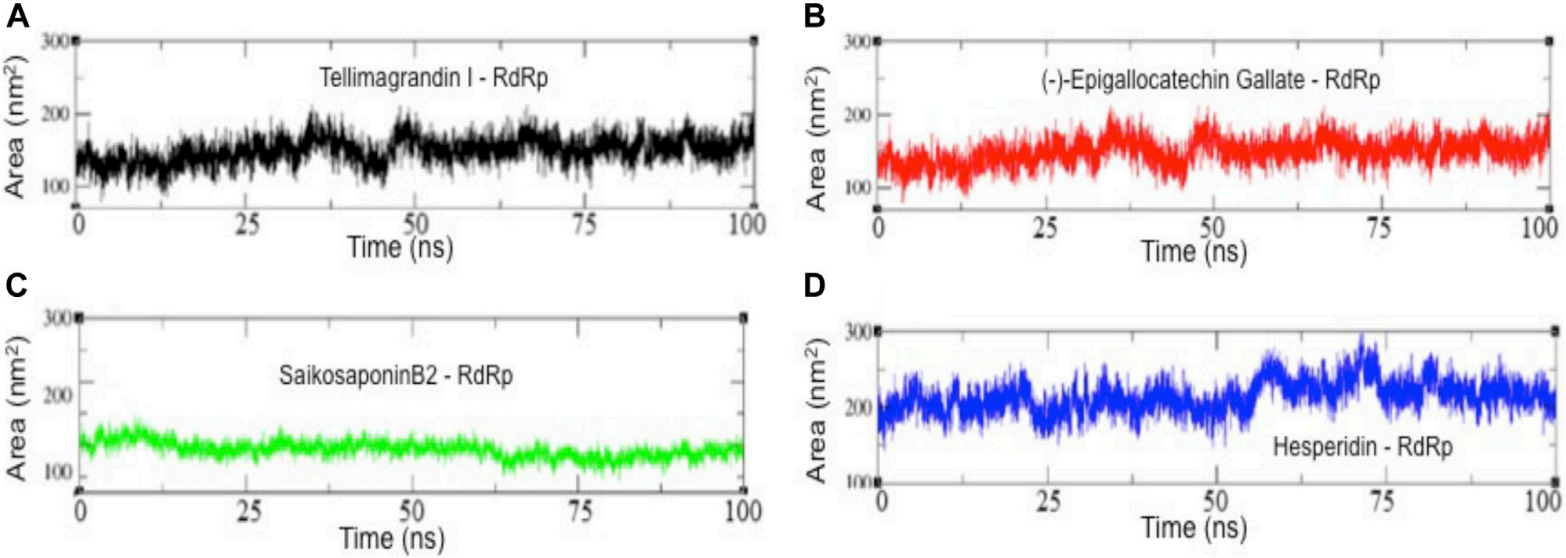
The solvent-accessible surface area (SASA) results of all four complexes. SASA plots of RdRp-Tellimagrandin I **(A)**, RdRp-(-)-Epigallocatechin Gallate **(B)**, RdRp-SaikosaponinB2 **(C)**, and RdRp-Hesperidin **(D)**. Molecular dynamic simulations were conducted for 100 ns?

We also examine the solvent-accessible surface area (SASA) of RdRp-inhibitor complexes, which provides the conformational concerning solvent around the protein (Figure 6). Results show a slight change or rather no change in the conformational dynamics of RdRp-Tellimagrandin I, RdRp-(-)-Epigallocatechin Gallate, and RdRp-SaikosaponinB2, which are converged around SASA values ∼150 Å^2^, respectively (Figures 6A–C respectively). However, the plot of RdRp-Hesperidin shows an increase in SASA value ∼80 Å^2^ during 0–5 ns, but it remains consistent up to 55 ns with the average fluctuation of ∼25–30 Å^2^ (Figure). However, the sharp drift of ∼50 Å^2^ can be seen at ∼60 ns, which gradually dropped at ∼75 ns, and the structure remains stable for ∼75–100 ns (Figure 6D).

Thus, the combined results of structural order parameters highlighted the more stable structural dynamics of RdRp complexed with Tellimagrandin I, SaikosaponinB2, and (-)-Epigallocatechin Gallate as compared to Hesperidin.

### MM-GBSA Assay

Finally, to evaluate the molecular binding of drug molecules with RdRp, the quantitative assessment of binding free energy (∆G_binding_) was carried out using MM-GBSA (Genheden and Ryde, 2015; Wang et al., 2019) on the conformational ensemble of protein-ligand complexes. Considering the convergence of MM/GBSA free energy estimates, only the last 20 ns of data were used for the analysis (Genheden and Ryde, 2015; Wang et al., 2019; Kumar et al., 2021). Results revealed that the binding affinities (∆G_binding_) of the selected drug molecules against RdRp range from −42.43 to −22.72 kcal/mol (Table 4). The non-bonded terms van der Waals energies (∆E_vdW_) are relatively more negative than the others from −64.57 to −37.35 kcal/mol, indicating that all four compounds have good hydrophobic contacts at the active site of RdRp (Table 4). However, the bonded terms electrostatic interactions (∆E_electrostatic_) ranges between −26.24 to −4.96 kcal/mol, which shows less contribution energy component, electrostatic in the relative stabilities of ligands (Table 4). Furthermore, the polar solvation energies (ΔE_GB_) act against the complexation, neutralized by bonded and non-bonded interactions. We noticed the higher contribution of ∆E_electrostatic_ = −26.24 kcal/mol and lower ∆E_electrostatic_ = −4.96 kcal/mol for drug molecules, SaikosaponinB2 and (-)-Epigallocatechin Gallate, respectively (Table 4). However, the higher contribution of ∆E_vdW_ = −39.95 kcal/mol results in the more favorable binding of (-)-Epigallocatechin Gallate, as compared to Hesperidin (∆E_vdW_ = −37.35) (Table 4). Among the four compounds, SaikosaponinB2 shows the best binding affinity (∆G_binding_ = −42.43 kcal/mol) for RdRp, whereas the least activity is noticed for the drug molecule, Hesperidin (−22.72 kcal/mol) (Table 4).

**TABLE 4:** Binding free energy (kcal/mol) approximation of drug molecules (Tellimagrandin I, SaikosaponinB2, Hesperidin, and (-)-Epigallocatechin Gallate) against RNA dependent RNA polymerase (RdRp).

Compounds	∆G_binding_	∆E_vdW_	∆E_electrostatic_	∆E_GB_	∆E_SURF_	∆G_gas_	∆G_solv_
Tellimagrandin I	−32.43 ± 3.06	−48.45 ± 2.65	−12.64 ± 4.14	34.41 ± 3.94	−5.75 ± 0.19	−61.10 ± 4.88	28.66 ± 3.93
SaikosaponinB2	−42.43 ± 3.25	−64.57 ± 2.33	−26.24 ± 3.16	56.38 ± 6.15	−7.99 ± 0.23	−90.81 ± 7.52	48.38 ± 6.11
Hesperidin	−22.72 ± 3.64	−37.35 ± 2.64	−24.56 ± 5.70	44.06 ± 5.04	−4.87 ± 0.29	−61.91 ± 6.26	39.19 ± 4.96
(-)-Epigallocatechin gallate	−24.17 ± 2.73	−39.95 ± 2.18	−4.96 ± 0.23	25.97 ± 5.95	−5.23 ± 0.36	−44.91 ± 6.76	20.73 ± 3.80

## Discussions

In this present study, RNA dependent RNA polymerase was taken as a drug target as this protein is essential for viral replication and transcription of SARS-CoV-2. The conserved RdRp catalytic motifs (A–E), the palm regions were taken as active sites. Plant products, mainly flavonoids, alkaloids, lactones, and terpenes, were considered during the investigation as inhibitors against the target protein. It has been earlier noted that natural products have traditionally provided the pharmaceutical industries with many vital leads to discover new drugs. Many compounds were isolated from a plant having an anti-viral activity (Lin et al., 1999; Cheng et al., 2004; Chang et al., 2005).

In this study, 248 natural compounds as ligands were selected. Four ligands possess an excellent binding affinity toward the target protein's active site depicting the lowest binding energy (Table 1), demonstrating their potentiality as inhibitors for SARS-CoV-2. The compound Tellimagrandin I depicted the best docking score of -9.6 kcal/mol (Table 1). The Druglike properties of the selected ligands were evaluated based on Lipinski parameters. Other than (-)-Epigallocatechin Gallate, all molecules violate the Lipinski rule as they have higher molecular weight. As a rule, it does not predict if a compound is pharmacologically active, and the already established antiviral activity in the previous study cannot be overlooked (Lin et al., 2014). Generally, a molecule showing a negative drug score is not considered a promising drug candidate; all four selected molecules show a favorable/positive drug score (Supplementary Table S2 and Supplementary Figures S5A–S5D); additionally, SaikosaponinB2 possess antiviral activity against HCOV-229 E (Cheng et al., 2006) and possesses a high activity with an IC50 value of 1.7 μmol/L (Cheng et al., 2006). Therefore, SaikosaponinB2 can be considered a potent inhibitor against COVID-19.

In the present study, hesperidin showed good drug scores, as shown in Supplementary Table S2 and Supplementary Figure S5D. This compound changes the immune system response by regulating interferons during influenza A virus infection and shows antiviral activity against influenza A virus (Randall and Goodbourn, 2008). This compound also exhibits antioxidant, anti-inflammatory, and lipid-lowering properties (Li and Schluesener, 2017) (-)-Epigallocatechin Gallate is one of the selected molecules favoring all the Lipinski rules with a good drug score (Supplementary Table S2 and Supplementary Figure S5B) (-)-Epigallocatechin Gallate had antiviral activity against the Hepatitis C virus with IC50 value 5 μg/L (Calland et al., 2012), Enterovirus 71 (Ho et al., 2009), and Zika virus (Carneiro et al., 2016). This polyphenol has antioxidant properties. Tellimagrandin I obtained from *Rosea rugosa* showed the highest binding affinity toward the target protein and possessed an excellent drug score (Supplementary Table S2 and Supplementary Figure S5A). Tellimagrandin I compound is also reported to have antiviral activity against the Hepatitis C virus (Tamura et al., 2010). Hence, Hesperidin (-)-Epigallocatechin Gallate, and Tellimagrandin I can also be considered potential drug candidates against SARS-CoV-2 RdRp protein.

In comparison, the two nucleoside analogs-favipiravir and remdesivir currently being evaluated in clinical trials for the treatment of COVID-19 scored lower −5.7 and −6.9 kcal/mol respectively against the RdRp (Table 1), thus enunciating the possibility of our proposed molecules to be a potent inhibitor against the SARS-CoV-19 RdRp protein.

The ligand-binding directly induced the structural changes needed to be accounted for the proteins to compute their binding free energies reliably, so we have followed this procedure.

A drug molecule’s efficacy depends on the spatial binding at the target protein’s active site and the protein-ligand complex’s structural stability (Luthra et al., 2009; Mishra et al., 2018; Majewski et al., 2019; Wang et al., 2020). Structure-based virtual screening is a reliable strategy to identify a potential inhibitor in the drug development process (Meng et al., 2011; Agrawal et al., 2019). The molecular docking using AutoDock allows our compounds to interact with our query protein in a single rigid conformation upon which score based ranking is determined. (Forli et al., 2016; Sulimov et al., 2019). Thus, to understand the protein-ligand molecular interactions, MD simulation can provide a comprehensive insight into protein-ligand interactions' structural stability and dynamic (Luthra et al., 2009; Prakash and Luthra, 2012; Wang et al., 2013; Panda et al., 2020). Thus, to determine the conformational dynamics and stability of protein-ligand complexes, MD simulations were carried out for 100 ns at the 300 K.

The structural order parameters evaluate the molecular stability of protein-ligand complexes. Figures 3A–D suggests that out of four complexes, the structure of RdRp- Tellimagrandin I and RdRp-(-)-Epigallocatechin Gallate quickly attain a stable equilibrium, and the RMSD trajectory observed consistent till the simulation ends at 100 ns? RMSD plot of RdRp-SaikosaponinB2 observed equilibrated around ∼2.7 Å, but the small drifts at ∼37 ns and ∼65 ns suggested the structural adjustment to accommodate the ligand at the active site protein, respectively (Figures 3A–C respectively). However, the structure of RdRp-Hesperidin shows a continuous rise in RMSD during the initial ∼0–25 ns and remains stable for ∼25–65 ns? A further slight increase in RMSD of ∼0.7 Å indicates relatively less stable conformational dynamics of complex structure with Hesperidin (Figure 3D).

Further, the structural compactness of RdRp-drug complexes determines by *R*_g_ analyses suggest the stable molecular interaction with all four compounds, which are stabilized in between the range of 31.25–33.50 Å (Figures 4A–D). However, the drifts of 0.20–0.40 Å can be seen during the initial stages of simulation in the R_g_ trajectory of Tellimagrandin I and SaikosaponinB2, which indicated the structural perturbation to accommodate the ligands. The average atomic fluctuations measured through RMSF plots suggest that all four RdRp-drug complexes show similar spatial binding patterns, which indicates that all four compounds remain well accommodated at the binding pocket of RdRp with favorable molecular interactions (Figures 5A–D). The hydrophobic interactions play a crucial role in determining the protein conformational dynamics, which ensure the structural stability of molecular interactions (Prakash et al., 2018b; Banerjee and Bagchi, 2020). Thus, we also investigated the SASA plots of all four complexes, suggesting no considerable changes in the conformational dynamics of Tellimagrandin I (-)-Epigallocatechin Gallate, and SaikosaponinB2 stabilized around SASA values 150 Å^2^, respectively (Figures 6A–C respectively). Whereas large deviation in SASA value approximately ∼50–80 Å^2^ reveals a relatively less stable structure of RdRp- Hesperidin complex (Figure 6D).

We further applied MM/GBSA free energy calculations to assess the thermodynamics stability of the RdRp complexed with drug molecules in terms of the binding free energy (Wang et al., 2019; Kumar et al., 2020). The MM/GBSA free energy calculation summarized in Table 4 provided clear evidence that the drug molecules were spatially stable at the active site of RdRp by van der Waals (∆E_vdW_) and electrostatic interactions (∆E_electrostatic_). Although the energy components, ΔE_GB_, act against the complexation, Van der Waals and electrostatic interactions mostly neutralize it. We can observe the more favorable molecular action of SaikosaponinB2 (∆G_binding_ = −42.43 kcal/mol), suggesting it as a potential candidate against RdRp in therapy against COVID-19 (Table 4).

## Conclusion

The present study explored the ligands' impacts, namely, Tellimagrandin I, SaikosaponinB2, Hesperidin (-)-Epigallocatechin Gallate’s molecular interactions and analyzed them as prospective drug candidates against the SARS-COV-2 RdRp protein. The screened molecules showed excellent docking scores, excellent pharmacokinetic profiles, MD simulation, and MM/GBSA profile. Moreover, these molecules cohere affirmatively with the predetermined amino acid residues present in the core palm region of the RdRp protein, thus inhibiting the viral gene replication and transcription. The ADMET results revealed excellent bioavailability and enzymatic inhibitory effect. Though the proposed molecules already have good IC50 values against different viruses, a further experimental analysis must be carried ahead to inspect its efficacy against the SARS-CoV-2. The binding free energy estimation using MM/PBSA assays revealed that selected-inhibitors: SaikosaponinB2, Tellimagrandin I, and (-)-Epigallocatechin Gallate possess better binding free energy and molecular affinity as compared to Hesperidin. Therefore, we proposed that selected molecules might be used as lead molecules in COVID-19 therapy.

The pharmacological profiling, docking analysis, MD simulation, MD trajectory, and MM/GBSA studies evaluated Saikosaponin B2 as a potent prospective drug candidate against the SARS-CoV-2 RdRp proteins that might inhibit duplication of COVID-19 virus, resulting in mitigating the disastrous global effects of the COVID-19 pandemic.

## Data Availability

The original contributions presented in the study are included in the article/Supplementary Material, further inquiries can be directed to the corresponding author.

## References

[B1] Agrawal P. Singh H. Srivastava H. K. Singh S. Kishore G. Raghava G. P. S. (2019). Benchmarking of different molecular docking methods for protein-peptide docking. BMC Bioinformatics. 19, 426. 10.1186/s12859-018-2449-y 30717654 PMC7394329

[B2] Azam S. S. Sarfaraz S. Abro A. (2014). Comparative modeling and virtual screening for the identification of novel inhibitors for myo-inositol-1-phosphate synthase. Mol. Biol. Rep. 41 (8), 5039–5052. 10.1007/s11033-014-3370-8 24752405

[B3] Bae E. A. Han M. J. Lee M. Kim D. H. (2000). *In Vitro* inhibitory effect of some flavonoids on rotavirus infectivity. Biol Pharm. Bull. 23 (9), 1122–1124. 10.1248/bpb.23.1122 10993220

[B4] Banerjee P. Bagchi B. (2020). Dynamical control by water at a molecular level in protein dimer association and dissociation. Proc. Natl. Acad. Sci. 117 (5), 2302–2308. 10.1073/pnas.1908379117 31969453 PMC7007538

[B5] Bhowmik D. Nandi R. Jagadeesan R. Kumar N. Prakash A. Kumar D. (2020). Identification of potential inhibitors against SARS-CoV-2 by targeting proteins responsible for envelope formation and virion assembly using docking based virtual screening, and pharmacokinetics approaches. Infect. Genet. Evol. 84, 104451. 10.1016/j.meegid.2020.104451 32640381 PMC7335633

[B6] Calland N. Albecka A. Belouzard S. Wychowski C. Duverlie G. Descamps V. (2012). (-)-Epigallocatechin-3-gallate is a new inhibitor of hepatitis C virus entry. Hepatology. 55 (3), 720–729. 10.1002/hep.24803 22105803

[B7] Carneiro B. M. Batista M. N. Braga A. C. S. Nogueira M. L. Rahal P. (2016). The green tea molecule EGCG inhibits Zika virus entry. Virology. 496, 215–218. 10.1016/j.virol.2016.06.012 27344138

[B8] Case D. A. Cheatham T. E. Darden T. Gohlke H. Luo R. Merz K. M. (2005). The Amber biomolecular simulation programs. J. Comput. Chem. 26 (16), 1668–1688. 10.1002/jcc.20290 16200636 PMC1989667

[B9] Chang J. S. Liu H. W. Wang K. C. Chen M. C. Chiang L. C. Hua Y. C. (2005). Ethanol extract of Polygonum cuspidatum inhibits hepatitis B virus in a stable HBV-producing cell line inhibits hepatitis B virus in a stable HBV‐producing cell line. Antivir. Res. 66 (1), 29–34. 10.1016/j.antiviral.2004.12.006 15781129

[B10] Cheng H. Y. Lin T. C. Yang C. M. Wang K. C. Lin L. T. Lin C. C. (2004). Putranjivain A from *Euphorbia* jolkini inhibits both virus entry and late stage replication of herpes simplex virus type 2 *in vitro* inhibits both virus entry and late stage replication of herpes simplex virus type 2. J. Antimicrob. Chemother. 53 (4), 577–583. 10.1093/jac/dkh136 14998984

[B11] Cheng P. W. Ng L. T. Chiang L. C. Lin C. C. (2006). Antiviral effects of saikosaponins on human coronavirus 229E *in vitro* . Clin. Exp. Pharmacol. Physiol. 33 (7), 612–616. 10.1111/j.1440-1681.2006.04415.x 16789928 PMC7162031

[B12] Doublié S. Ellenberger T. (1998). The mechanism of action of T7 DNA polymerase. Curr. Opin. Struct. Biol. 8 (6), 704–712. 10.1016/s0959-440x(98)80089-4 9914251

[B13] Douglas E. V. Blundell T. L. David B. A. (2015). pkCSM: predicting small-molecule pharmacokinetic and toxicity properties using graph-based signature. J. Med. Chem. 58 (9), 4066–4072. 10.1021/acs.jmedchem.5b00104 25860834 PMC4434528

[B14] Elfiky A. A. Ismail A. M. (2018). Molecular docking revealed the binding of nucleotide/side inhibitors to Zika viral polymerase solved structures. SAR QSAR Environ. Res. 29 (5), 409–418. 10.1080/1062936X/2018.1454981 29652194

[B15] Elfiky A. A. Azzam E. B. (2020). Novel Guanosine Derivatives against MERS CoV polymerase: an in silico perspective. J. Biomol. Struct. Dyn., 1–12. 10.1080/07391102/2020.1758789 PMC7189410 32306854

[B16] Essmann U. Perera L. Berkowitz M. L. Darden T. Lee H. Pedersen L. G. (1995). A smooth particle mesh Ewald method. J. Chem. Phys. 103, 8577–8593. 10.1063/1.470117

[B17] Forli S. Huey R. Pique M. E. Sanner M. F. Goodsell D. S. Olson A. J. (2016). Computational protein-ligand docking and virtual drug screening with the AutoDock suite. Nat. Protoc. 11 (5), 905–919. 10.1038/nprot.2016.051 27077332 PMC4868550

[B18] Gao Y. Yan L. Huang Y. Liu F. Zhao Y. Cao L. (2020). Structure of the RNA-dependent RNA polymerase from COVID-19 virus. Science. 368 (6492), 779–782. 10.1126/science.abb7498 32277040 PMC7164392

[B19] Genheden S. Ryde U. (2015). The MM/PBSA and MM/GBSA methods to estimate ligand-binding affinities. Expert Opin. Drug Discov. 10 (5), 449–461. 10.1517/17460441.2015.1032936 25835573 PMC4487606

[B20] Gorbalenya A. E. Pringle F. M. Zeddam J. L. Luke B. T. Cameron C. E. Kalmakoff J. (2002). The palm subdomain-based active site is internally permuted in viral RNA-dependent RNA polymerases of an ancient lineage. J. Mol. Biol. 324 (1), 47–62. 10.1016/s0022-2836(02)01033-1 12421558 PMC7127740

[B21] Ho H. Y. Cheng M. L. Weng S. F. Leu Y. L. Chiu D. T. (2009). Antiviral effect of epigallocatechin gallate on enterovirus 71. J. Agric. Food Chem. 57 (14), 6140–6147. 10.1021/jf901128u 19537794

[B22] Ibrahim I. M. Abdelmalek D. H. Elshahat M. E. Elfiky A. A. (2020). COVID-19 Spike-host cell receptor GRP78 binding site prediction. J. Infect. 80 (5), 554–562. 10.1016/j.jinf.2020.02.026 32169481 PMC7102553

[B23] Jorgensen W. L. Chandrasekhar J. Madura J. D. Impey R. W. Klein M. L. (1983). Comparison of simple potential functions for simulating liquid water. J. Chem. Phys. 79, 926–935. 10.1063/1.445869

[B24] Krieger E. Joo K. Lee J. Lee J. Raman S. Thompson J. (2009). Improving physical realism, stereochemistry, and side-chain accuracy in homology modeling: four approaches that performed well in CASP8. Proteins. 77 (Suppl. 9), 114–122. 10.1002/prot.22570 19768677 PMC2922016

[B25] Kumar N. Srivastava R. Prakash A. Lynn A. M. (2020). Structure-based virtual screening, molecular dynamics simulation and MM-PBSA toward identifying the inhibitors for two-component regulatory system protein NarL of *Mycobacterium Tuberculosis* . J. Biomol. Struct. Dyn. 38, 3396–3410. 10.1080/07391102.2019.1657499 31422761

[B26] Laskowski R. A. MacArthur M. W. Moss D. S. Thornton J. M. (1993). PROCHECK: a program to check the stereo chemical quality of protein structures. J. Appl. Crystallogr. 26 (2), 283–291. 10.1107/S0021889892009944

[B27] Li C. Schluesener H. (2017). Health-promoting effects of the citrus flavanone hesperidin. Crit. Rev. Food Sci. Nutr. 57 (3), 613–631. 10.1080/10408398.2014.906382 25675136

[B28] Lill M. A. Danielson M. L. (2011). Computer-aided drug design platform using PyMOL. J. Comput. Aided Mol. Des. 25 (1), 13–19. 10.1007/s10822-010-9395-8 21053052

[B29] Lin L. T. Hsu W. C. Lin C. C. (2014). Antiviral natural products and herbal medicines. J. Tradit Complement. Med. 4 (1), 24–35. 10.4103/2225-4110.124335 24872930 PMC4032839

[B30] Lin Y. M. Flavin M. T. Schure R. Chen F. C. Sidwell R. Barnard D. L. (1999). Antiviral activities of biflavonoids. Planta Med. 65 (2), 120–125. 10.1055/s-1999-13971 10193201

[B31] Lipinski C. A. (2014). Lead- and drug-like compounds: the rule-of-five revolution. Drug Discov. Today Technol. 1 (4), 337–341. 10.1016/j.ddtec.2004.11.007 24981612

[B32] Luthra P. M. Kumar R. Prakash A. (2009). Demethoxycurcumin induces Bcl-2 mediated G2/M arrest and apoptosis in human glioma U87 cells. Biochem. Biophys. Res. Commun. 384 (4), 420–425. 10.1016/j.bbrc.2009.04.149 19422808

[B33] Lythgoe M. P. Middleton P. (2020). Ongoing clinical trials for the management of the COVID-19 pandemic. Trends Pharmacol. Sci. 41 (6), 363–382. 10.1016/j.tips.2020.03.006 32291112 PMC7144665

[B34] Maier J. A. Martinez C. Kasavajhala K. Wickstrom L. Hauser K. E. Simmerling C. (2015). ff14SB: improving the accuracy of protein side chain and backbone parameters from ff99SB. J. Chem. Theor. Comput. 11 (8), 3696–3713. 10.1021/acs.jctc.5b00255 PMC4821407 26574453

[B35] Majewski M. Ruiz-Carmona S. Barril X. (2019). An investigation of structural stability in protein-ligand complexes reveals the balance between order and disorder. Commun. Chem. 2, 110. 10.1038/s42004-019-0205-5

[B36] Meng X. Y. Zhang H. X. Mezei M. Cui M. (2011). Molecular docking: a powerful approach for structure-based drug discovery. Curr. Comput. Aided Drug Des. 7 (2), 146–157. 10.2174/157340911795677602 21534921 PMC3151162

[B37] Mishra C. B. Kumari S. Prakash A. Yadav R. Tiwari A. K. Pandey P. (2018). Discovery of novel Methylsulfonyl phenyl derivatives as potent human Cyclooxygenase-2 inhibitors with effective anticonvulsant action: design, synthesis, in-silico, *in-vitro* and *in-vivo* evaluation. Eur. J. Med. Chem. 151, 520–532. 10.1016/j.ejmech.2018.04.007 29655084

[B38] Niranjan Kumar N. Srivastava R. Prakash A. Lynn A. M. (2021). Virtual screening and free energy estimation for identifying *Mycobacterium tuberculosis* flavoenzyme DprE1 inhibitors. J. Mol. Graph Model. 102, 107770. 10.1016/j.jmgm.2020.107770 33065513

[B39] Nygaard M. Kragelund B. B. Papaleo E. Lindorff-Larsen K. (2017). An efficient method for estimating the hydrodynamic radius of disordered protein conformations. Biophys. J. 113 (3), 550–557. 10.1016/j.bpj.2017.06.042 28793210 PMC5550300

[B40] Panda P. K. Arul M. N. Patel P. Verma S. K. Luo W. Rubahn H. G. (2020). Structure-based drug designing and immunoinformatics approach for SARS-CoV-2. Sci. Adv. 6 (28), eabb8097. 10.1126/sciadv.abb8097 32691011 PMC7319274

[B41] Prakash A. Dixit G. Meena N. K. Singh R. Vishwakarma P. Mishra S. (2018a). Elucidation of stable intermediates in urea-induced unfolding pathway of human carbonic anhydrase IX. J. Biomol. Struct. Dyn. 36 (9), 2391–2406. 10.1080/07391102.2017.1355847 28705076

[B42] Prakash A. Luthra P. M. (2012). Insilico study of the A(2A)R-D (2)R kinetics and interfacial contact surface for heteromerization. Amino Acids. 43, 1451–1464. 10.1007/s00726-012-1218-x 22278740

[B43] Prakash A. Kumar V. Meena N. K. Lynn A. M. (2018b). Elucidation of the structural stability and dynamics of heterogeneous intermediate ensembles in unfolding pathway of the N-terminal domain of TDP-43. RSC Adv. 8, 19835–19845. 10.1039/C8RA03368D PMC9088055 35548664

[B44] Randall R. E. Goodbourn S. (2008). Interferons and viruses: an interplay between induction, signalling, antiviral responses and virus countermeasures. J. Gen. Virol. 89 (Pt 1), 1–47. 10.1099/vir.0.83391-0 18089727

[B45] Ryckaert J. P. Ciccotti G. Berendsen H. J. C. (1977). Numerical integration of the cartesian equations of motion of a system with constraints: molecular dynamics of n-alkanes. J. Comput. Phys. 23, 327–341. 10.1016/0021-9991(77)90098-5

[B46] Shu B. Gong P. (2016). Structural basis of viral RNA-dependent RNA polymerase catalysis and translocation. Proc. Natl. Acad. Sci. USA. 113 (28), E4005–E4014. 10.1073/pnas.1602591113 27339134 PMC4948327

[B47] Singh S. Fulbabu S. k. Sonawane A. Kar P. Sadhukhan S. (2020). Plant-derived natural polyphenols as potential antiviral drugs against SARS-CoV-2 via RNA‐dependent RNA polymerase (RdRp) inhibition: an in-silico analysis. J. Biomol. Struct. Dyn. 28 (7), 1–16. 10.1080/07391102.2020.1796810 PMC7441777 32720577

[B48] Sinha M. Jagadeesan R. Kumar N. Saha S. Kothandan G. Kumar D. (2020). In-silico studies on Myo inositol-1-phosphate synthase of Leishmania donovani in search of anti-leishmaniasis. J. Biomol. Struct. Dyn., 1–14. 10.1080/07391102.2020.1847194 33200690

[B49] Sulimov V. B. Kutov D. C. Sulimov A. V. (2019). Advances in docking. Curr. Med. Chem. 26 (42), 7555–7580. 10.2174/0929867325666180904115000 30182836

[B50] Tamura S. Yang G. M. Yasueda N. Matsuura Y. Komoda Y. Murakami N. (2010). Tellimagrandin I, HCV invasion inhibitor from rosae rugosae flos. Bioorg. Med. Chem. Lett. 20 (5), 1598–1600. 10.1016/j.bmcl.2010.01.084 20144544

[B51] Tian W. Chen C. Lei X. Zhao J. Liang J. (2018). CASTp 3.0: computed atlas of surface topography of proteins. Nucleic Acids Res. 46(W1), W363–W367. 10.1093/nar/gky473 29860391 PMC6031066

[B52] Trott O. Olson A. J. (2010). AutoDock Vina: improving the speed and accuracy of docking with a new scoring function, efficient optimization, and multithreading. J. Comput. Chem. 31 (2), 455–461. 10.1002/jcc.21334 19499576 PMC3041641

[B53] Venkataraman S. Prasad B. V. L. S. Selvarajan R. (2018). RNA dependent RNA polymerases: insights from structure, function and evolution. Viruses. 10 (2), 76. 10.3390/v10020076 PMC5850383 29439438

[B54] Wallace A. C. Laskowski R. A. Thornton J. M. (1995). LIGPLOT: a program to generate schematic diagrams of protein-ligand interactions. Protein Eng. 8 (2), 127–134. 10.1093/protein/8.2.127 7630882

[B55] Walls A. C. Park Y. J. Tortorici M. A. Wall A. McGuire A. T. Veesler D. (2020). Structure, function, and antigenicity of the SARS-CoV-2 spike glycoprotein. Cell. 181 (2), 281–e6. 10.1016/j.cell.2020.02.058 32155444 PMC7102599

[B56] Wang D. D. Ou-Yang L. Xie H. Zhu M. Yan H. (2020). Predicting the impacts of mutations on protein-ligand binding affinity based on molecular dynamics simulations and machine learning methods. Comput. Struct. Biotechnol. J. 18, 439–454. 10.1016/j.csbj.2020.02.007 32153730 PMC7052406

[B57] Wang E. Sun H. Wang J. Wang Z. Liu H. Zhang J. Z. H. (2019). End-point binding free energy calculation with MM/PBSA and MM/GBSA: strategies and applications in drug design. Chem. Rev. 119 (16), 9478–9508. 10.1021/acs.chemrev.9b00055 31244000

[B58] Wang F. Sambandan D. Halder R. Wang J. Batt S. M. Weinrick B. (2013). Identification of a small molecule with activity against drug-resistant and persistent *tuberculosis* . Proc. Natl. Acad. Sci. United States. 110 (27), E2510–E2517. 10.1073/pnas.1309171110 PMC3703973 23776209

[B59] Wang J. Wang W. Kollman P. A. Case D. A. (2006). Automatic atom type and bond type perception in molecular mechanical calculations. J. Mol. Graph Model. 25 (2), 247–260. 10.1016/j.jmgm.2005.12.005 16458552

[B60] Waterhouse A. Bertoni M. Bienert S. Studer G. Tauriello G. Gumienny R. (2018). SWISS-MODEL: homology modelling of protein structures and complexes. Nucleic Acids Res. 46 (W1), W296–W303. 10.1093/nar/gky427 29788355 PMC6030848

[B61] Wiederstein M. Sippl M. J. (2007). ProSA-web: interactive web service for the recognition of errors in three-dimensional structures of proteins. Nucleic Acids Res. 35, W407–W410. 10.1093/nar/gkm290 17517781 PMC1933241

[B62] Zhou P. Yang X. L. Wang X. G. Hu B. Zhang L. Zhang W. (2020). A pneumonia outbreak associated with a new coronavirus of probable bat origin. Nature. 579 (7798), 270–273. 10.1038/s41586-020-2012-7 32015507 PMC7095418

