# Supplementary material for: Discovering Potential RNA Dependent RNA Polymerase Inhibitors as Prospective Drugs Against COVID-19: An in silico Approach
Source: Front Pharmacol. 2021 Feb 26;12:634047. doi: 10.3389/fphar.2021.634047 (PMC7952625; doi:10.3389/fphar.2021.634047)
Supplement: Supplementary file 1 [file datasheet1.pdf]

Supplementary data

“Discovering potential RNA dependent RNA polymerase inhibitors as prospective drugs against COVID-19: an in silico approach."

Satabdi saha[1§], Rajat Nandi[1§], Poonam Vishwakarma[2], Amresh Prakash[3] and Diwakar Kumar[1]*

[1]Department of Microbiology, Assam University, Silchar-788011, Assam, India

[2]School of Computational and Integrative Sciences, Jawaharlal Nehru University, New Delhi-110067

[3]Amity Institute of Integrative Sciences and Health, Amity University Haryana, Gurgaon-122413, India

[§]Equally contributed as the first author

***Corresponding Author:**

Diwakar Kumar, PhD.,
Department of Microbiology, Assam University, Silchar-788011, Assam, India
E-mail: diwakar11@gmail.com
Tel: +91-8134080245

Supplementary Tables

Table S1. Docking table of the ligands.

Sl. No.	Name	Smiles	PubChem CID	Docking Score
1	Saikosaponin A	CC1C(C(C(C(O1)OC2CCC3(C(C2(C)CO)CCC4(C3C=CC56C4(CC(C7(C5CC(CC7)(C)C)CO6)O)C)C)C)O)OC8C(C(C(C(O8)CO)O)O)O)O	167928	-5.1
2	SaikosaponinB2	C1C(C(C(C(O1)OC2CCC3(C(C2(C)CO)CCC4(C3C=CC5=C6CC(CCC6(C(CC54C)O)CO)(C)C)C)C)O)OC7C(C(C(C(O7)CO)O)O)O)O)O	21637642	-8.9
3	Saikosaponin D	CC1C(C(C(C(O1)OC2CCC3(C(C2(C)CO)CCC4(C3C=CC56C4(CC(C7(C5CC(CC7)(C)C)CO6)O)C)C)C)O)OC8C(C(C(C(O8)CO)O)O)O)O	107793	-9.2
4	Amentoflavone	C1=CC(=CC=C1C2=CC(=O)C3=C(O2)C(=C(C=C3O)O)C4=C(C=CC(=C4)C5=CC(=O)C6=C(C=C(C=C6O5)O)O)O)O	5281600	-5.1
5	Lycorine hydrochloride	C1CN2CC3=CC4=C(C=C3C5C2C1=CC(C5O)O)OCO4.Cl	164943	-6.0
6	Lycorine	C1CN2CC3=CC4=C(C=C3C5C2C1=CC(C5O)O)OCO4	72378	-5.8
7	Scutellarein	C1=CC(=CC=C1C2=CC(=O)C3=C(O2)C=C(C(=C3O)O)O)O	5281697	-7.2
8	Scutellarin	C1=CC(=CC=C1C2=CC(=O)C3=C(C(=C(C=C3O2)OC4C(C(C(C(O4)C(=O)O)O)O)O)O)O)O	185617	-6.1
9	Linalool	CC(=CCCC(C)(C=C)O)C	6549	-5.8
10	Ursolic Acid	CC1CCC2(CCC3(C(=CCC4C3(CCC5C4(CCC(C5(C)C)O)C)C)C2C1C)C)C(=O)O	64945	-7.4
11	Chebulagic acid	C1C2C3C(C(C(O2)OC(=O)C4=CC(=C(C(=C4)O)O)O)OC(=O)C5=CC(=C(C6=C5C(C(C(=O)O3)CC(=O)O)C(C(=O)O6)O)O)O)OC(=O)C7=CC(=C(C(=C7C8=C(C(=C(C=C8C(=O)O1)O)O)O)O)O)O	442674	-5.5
12	Raoulic acid	CC(=CCC1CC(CCC2(C(CCC1=C)CC2C(=C)C)C)C(=C)C(=O)O)C	49771359	-4.8
13	Punicalagin	C1C2C(C3C(C(O2)O)OC(=O)C4=CC(=C(C(=C4C5=C(C(=C(C=C5C(=O)O3)O)O)O)O)O)O)OC(=O)C6=CC(=C(C(=C6C7=C(C(=C8C9=C7C(=O)OC2=C(C(=C(C3=C(C(=C(C=C3C(=O)O1)O)O)O)C(=C92)C(=O)O8)O)O)O)O)O)O)O	44584733	-5.2
14	Gallic acid	C1=C(C=C(C(=C1O)O)O)C(=O)O	370	-6.6
15	Epigallocatechin	C1C(C(OC2=CC(=CC(=C21)O)O)C3=CC(=C(C(=C3)O)O)O)O	72277	-7.6
16	(-)-Epigallocatechin Gallate	C1C(C(OC2=CC(=CC(=C21)O)O)C3=CC(=C(C(=C3)O)O)O)OC(=O)C4=CC(=C(C(=C4)O)O)O	65064	-8.1
17	Isochlorogenic acid C	C1C(C(C(CC1(C(=O)O)O)O)OC(=O)C=CC2=CC(=C(C=C2)O)O)OC(=O)C=CC3=CC(=C(C=C3)O)O)O	6474309	-5.3

18	Apigenin	C1=CC(=CC=C1C2=CC(=O)C3=C(C=C(C=C3O2)O)O)O	5280443	-5.6
19	Quercetin	C1=CC(=C(C=C1C2=C(C(=O)C3=C(C=C(C=C3O2)O)O)O)O)O	5280343	-6.2
20	Narasin	CCC(C1C(CC(C(O1)C(C)C(C(C)C(=O)C(CC)C2C(CC(C3(O2)C=CC(C4(O3)CCC(O4)(C)C5CCC(C(O5)C)(CC)O)O)C)C)O)C)C)C(=O)O	65452	-5.1
21	Naringin	CC1C(C(C(C(O1)OC2C(C(C(OC2OC3=CC(=C4C(=O)CC(OC4=C3)C5=CC=C(C=C5)O)O)CO)O)O)O)O)O	442428	-7.7
22	Silymarin	COC1=C(C=CC(=C1)C2C(OC3=C(O2)C=C(C=C3)C4C(C(=O)C5=C(C=C(C=C5O4)O)O)O)CO)O	7073228	-5.7
23	Ladanein	COC1=CC=C(C=C1)C2=CC(=O)C3=C(C(=C(C=C3O2)OC)O)O	3084066	-4.2
24	Tellimagrandin I	C1C2C(C(C(C(O2)O)OC(=O)C3=CC(=C(C(=C3)O)O)O)OC(=O)C4=CC(=C(C(=C4)O)O)O)OC(=O)C5=CC(=C(C(=C5C6=C(C(=C(C=C6C(=O)O1)O)O)O)O)O)O	442690	-9.6
25	Hippomanin A	C1C2C(C(C(C(O2)O)OC(=O)C3=CC(=C(C(=C3)O)O)O)O)OC(=O)C4=CC(=C(C(=C4C5=C(C(=C(C=C5C(=O)O1)O)O)O)O)O)O	323958	-5.2
26	Geraniin	C1C2C3C(C(C(O2)OC(=O)C4=CC(=C(C(=C4)O)O)O)OC(=O)C5=CC(=C(C6=C5C7C(=CC(=O)C(C7(O)O)(O6)O)C(=O)O3)O)O)OC(=O)C8=CC(=C(C(=C8C9=C(C(=C(C=C9C(=O)O1)O)O)O)O)O)O	3001497	-7.8
27	Excoecarianin	C1C2C3C(C(C(O2)OC(=O)C4=CC(=C(C(=C4)O)O)O)OC(=O)C5=CC(=C(C6=C5C7C(=CC(=O)C(C7(O)O)(O6)O)C(=O)O3)O)O)OC(=O)C8=C(C(=C(C(=C8)C9=C(C(=C(C=C9C(=O)O1)OC1=C(C(=C(C=C1C(=O)OC1C2C(C(COC(=O)C3=CC(=C(C(=C3C3=C(C(=C(C=C3C(=O)O2)O)O)O)O)O)O)O)OC1OC(=O)C1=CC(=C(C(=C1)O)O)O)OC(=O)C1=CC(=C(C(=C1)O)O)O)O)O)O)O)O)O)O)O	49774003	-8.1
28	Procyanidin	C1C(C(OC2=CC(=CC(=C21)O)O)C3=CC(=C(C=C3)O)O)OC4(C(C(C5=C(C=C(C=C5O4)O)O)O)O)C6=CC(=C(C=C6)O)O	107876	-5.4
29	Proanthocyanidin	COC1=C(C=C(C=C1O)C2C(CC3=C(O2)C(=C(C=C3O)O)C4C(C(OC5=CC(=CC(=C45)O)O)C6=CC=C(C=C6)O)O)O)O	108065	-6.3
30	Glucoevatromonoside	CC1C(C(CC(O1)OC2CCC3(C(C2)CCC4C3CCC5(C4(CCC5C6=CC(=O)OC6)O)C)C)O)OC7C(C(C(C(O7)CO)O)O)O	15137997	-7.2
31	Coumarin	C1=CC=C2C(=C1)C=CC(=O)O2	323	-8.1
32	Spironolactone	CC(=O)SC1CC2=CC(=O)CCC2(C3C1C4CCC5(C4(CC3)C)CCC(=O)O5)C	5833	-7.8
33	Spirooliganone B	CC(C)C12CCC3(C1C2)CCC4=C(O3)C5(CC(C(O5)(C)C)O)C=C(C4=O)CC=C	122396353	-7.5
34	Chalcone	C1=CC=C(C=C1)C=CC(=O)C2=CC=CC=C2	637760	-6.6
35	Licochalcone A	CC(C)(C=C)C1=C(C=C(C(=C1)C=CC(=O)C2=CC=C(C=C2)O)OC)O	5318998	-7.8
36	Uncinoside A	CC1=CC(=O)C2=C(O1)C(=C(C(=C2O)C)OC3C(C(C(C(O3)CO)O)O)O)C	10022766	-7.4
37	Uncinoside B	CC1=CC(=O)C2=C(C(=C(C(=C2O1)C)OC3C(C(C(C(O3)CO)O)O)O)C)OC(=O)C	11743072	-5.8

38	Genkwanol B	C1C(C(OC2=C1C(=O)C=C3C24C(C(O3)C5=CC=C(C=C5)O)(C(=O)C6=C(C=C(C=C6O4)O)O)O)C7=CC=C(C=C7)O)O	15735849	-6.7
39	Genkwanol C	C1C(C(OC2=C1C(=O)C=C3C24C(C(O3)C5=CC=C(C=C5)O)(C(=O)C6=C(C=C(C=C6O4)O)O)O)C7=CC=C(C=C7)O)O	131676073	-7.2
40	Cimifugin	CC(C)(C1CC2=C(O1)C=C3C(=C2OC)C(=O)C=C(O3)CO)O	441960	-6.4
41	Butein	C1=CC(=C(C=C1C=CC(=O)C2=C(C=C(C=C2)O)O)O)O	5281222	-5.4
42	Fustin	C1=CC(=C(C=C1C2C(C(=O)C3=C(O2)C=C(C=C3)O)O)O)O	5317435	-4.8
43	Morin	C1=CC(=C(C=C1O)O)C2=C(C(=O)C3=C(C=C(C=C3O2)O)O)O	5281670	-6.7
44	Galangin	C1=CC=C(C=C1)C2=C(C(=O)C3=C(C=C(C=C3O2)O)O)O	5281616	-4.3
45	Hispidulin	COC1=C(C2=C(C=C1O)OC(=CC2=O)C3=CC=C(C=C3)O)O	5281628	-6.3
46	Luteolin	C1=CC(=C(C=C1C2=CC(=O)C3=C(C=C(C=C3O2)O)O)O)O	5280445	-4.8
47	Paeonol	CC(=O)C1=C(C=C(C=C1)OC)O	11092	-5.7
48	Syringic acid	COC1=CC(=CC(=C1O)OC)C(=O)O	10742	-7.0
49	Piceatannol	C1=CC(=C(C=C1C=CC2=CC(=CC(=C2)O)O)O)O	667639	-4.2
50	Danshensu	C1=CC(=C(C=C1CC(C(=O)O)O)O)O	11600642	-6.9
51	Pinobanksin	C1=CC=C(C=C1)C2C(C(=O)C3=C(C=C(C=C3O2)O)O)O	73202	-3-2
52	Ferulic acid	COC1=C(C=CC(=C1)C=CC(=O)O)O	445858	-6.5
53	Pyrogallol	C1=CC(=C(C(=C1)O)O)O	1057	-4-8
54	Nobiletin	COC1=C(C=C(C=C1)C2=CC(=O)C3=C(O2)C(=C(C(=C3OC)OC)OC)OC)OC	72344	-7.1
55	Purpurin	C1=CC=C2C(=C1)C(=O)C3=C(C2=O)C(=C(C=C3O)O)O	6683	-4.8
56	Gossypetin	C1=CC(=C(C=C1C2=C(C(=O)C3=C(O2)C(=C(C=C3O)O)O)O)O)O	5280647	-6.2
57	Tangeritin	COC1=CC=C(C=C1)C2=CC(=O)C3=C(O2)C(=C(C(=C3OC)OC)OC)OC	68077	-4.5
58	Peonidin	COC1=C(C=CC(=C1)C2=[O+]C3=CC(=CC(=C3C=C2O)O)O)O	441773	-6.5
59	Wogonin	COC1=C(C=C(C2=C1OC(=CC2=O)C3=CC=CC=C3)O)O	5281703	-4.2
60	Pendunculagin	C1C2C(C3C(C(O2)O)OC(=O)C4=CC(=C(C(=C4C5=C(C(=C(C=C5C(=O)O3)O)O)O)O)O)O)OC(=O)C6=CC(=C(C(=C6C7=C(C(=C(C=C7C(=O)O1)O)O)O)O)O)O	442688	-5.2
61	Punigluconin	C1C(C(OC(=O)C2=CC(=C(C(=C2C3=C(C(=C(C=C3C(=O)O1)O)O)O)O)O)O)C(C(C(=O)O)OC(=O)C4=CC(=C(C(=C4)O)O)O)OC(=O)C5=CC(=C(C(=C5)O)O)O)O	21637585	-4.3

62	Tellimagrandin II	C1C2C(C(C(C(O2)OC(=O)C3=CC(=C(C(=C3)O)O)O)OC(=O)C4=CC(=C(C(=C4)O)O)O)OC(=O)C5=CC(=C(C(=C5)O)O)O)OC(=O)C6=CC(=C(C(=C6C7=C(C(=C(C=C7C(=O)O1)O)O)O)O)O)O	151590	-5.2
63	Castalagin	C1C2C(C3C4C(C5=C(C(=C(C(=C5C(=O)O4)C6=C(C(=C(C(=C6C(=O)O3)C7=C(C(=C(C=C7C(=O)O2)O)O)O)O)O)O)O)O)O)O)OC(=O)C8=CC(=C(C(=C8C9=C(C(=C(C=C9C(=O)O1)O)O)O)O)O)O	168165	-4.8
64	Terflavin B	C1=C(C=C(C(=C1O)O)O)C(=O)OCC2C(C(C(C(O2)O)O)O)OC(=O)C3=CC(=C(C(=C3C4=C(C(=C5C6=C4C(=O)OC7=C6C(=CC(=C7O)O)C(=O)O5)O)O)O)O)O	44584734	-6.7
65	Roburin A	C1C2C(C3C4C(C5=C(C(=C(C(=C5C(=O)O4)C6=C(C(=C(C(=C6C(=O)O3)C7=C(C(=C(C=C7C(=O)O2)O)O)O)O)O)O)O)O)O)O)C8=C(C(=C(C9=C8C(=O)OCC2C(C3C4C(C5=C(C(=C(C(=C5C(=O)O4)C4=C(C(=C(C(=C4C(=O)O3)C3=C(C(=C(C=C3C(=O)O2)O)O)O)O)O)O)O)O)O)O)OC(=O)C2=CC(=C(C(=C29)O)O)O)O)O)O)OC(=O)C2=CC(=C(C(=C2C2=C(C(=C(C=C2C(=O)O1)O)O)O)O)O)O	101670390	-5.8
66	Vescalagin	C1C2C(C3C4C(C5=C(C(=C(C(=C5C(=O)O4)C6=C(C(=C(C(=C6C(=O)O3)C7=C(C(=C(C=C7C(=O)O2)O)O)O)O)O)O)O)O)O)O)OC(=O)C8=CC(=C(C(=C8C9=C(C(=C(C=C9C(=O)O1)O)O)O)O)O)O	168165	-4.3
67	Castalin	C1=C2C(=C(C(=C1O)O)O)C3=C4C(=C(C(=C3O)O)O)C5=C6C(=C(C(=C5O)O)O)C(C(C(C(C(OC2=O)CO)O)OC4=O)OC6=O)O	99973	-5.6
68	Casuarinin	C1C(C(OC(=O)C2=CC(=C(C(=C2C3=C(C(=C(C=C3C(=O)O1)O)O)O)O)O)O)C4C5C(C6=C(C(=C(C(=C6C(=O)O5)C7=C(C(=C(C=C7C(=O)O4)O)O)O)O)O)O)O)OC(=O)C8=CC(=C(C(=C8)O)O)O	442673	-6.1
69	Grandinin	C1C2C(C3C4C(C5=C(C(=C(C(=C5C(=O)O4)C6=C(C(=C(C(=C6C(=O)O3)C7=C(C(=C(C=C7C(=O)O2)O)O)O)O)O)O)O)O)O)O)C8(C(C(C(O8)CO)O)O)O)OC(=O)C9=CC(=C(C(=C9C2=C(C(=C(C=C2C(=O)O1)O)O)O)O)O)O	492392	-5.8
70	Rosmarinic acid	C1=CC(=C(C=C1CC(C(=O)O)OC(=O)C=CC2=CC(=C(C=C2)O)O)O)O	5281792	-4.5
71	Ellagic acid	C1=C2C3=C(C(=C1O)O)OC(=O)C4=CC(=C(C(=C43)OC2=O)O)O	5281855	-3.2
72	Punicalin	C1C2C(C(C(C(O2)O)O)O)OC(=O)C3=CC(=C(C(=C3C4=C(C(=C5C6=C4C(=O)OC7=C(C(=C(C8=C(C(=C(C=C8C(=O)O1)O)O)O)C(=C67)C(=O)O5)O)O)O)O)O)O	5388496	-5.6
73	Daidzein	C1=CC(=CC=C1C2=COC3=C(C2=O)C=CC(=C3)O)O	5281708	-7.6
74	Equol	C1C(COC2=C1C=CC(=C2)O)C3=CC=C(C=C3)O	91469	-6.1

75	Raspberry ellagitannin	C1C2C(C3C(C(O2)OC4=C(C(=C(C5=C4C(=O)OC6C(COC(=O)C7=CC(=C(C(=C75)O)O)O)OC(C8C6OC(=O)C9=CC(=C(C(=C9C2=C(C(=C(C=C2C(=O)O8)O)O)O)O)O)O)OC(=O)C2=CC(=C(C(=C2)OC2=C(C(=C(C4=C2C(=O)OC2C(COC(=O)C5=CC(=C(C(=C54)O)O)O)OC(C4C2OC(=O)C2=CC(=C(C(=C2C2=C(C(=C(C=C2C(=O)O4)O)O)O)O)O)O)OC(=O)C2=CC(=C(C(=C2)O)O)O)O)O)O)O)O)O)O)O)OC(=O)C2=CC(=C(C(=C2C2=C(C(=C(C=C2C(=O)O3)O)O)O)O)O)O)OC(=O)C2=CC(=C(C(=C2C2=C(C(=C(C=C2C(=O)O1)O)O)O)O)O)O	71308261	-6.2
76	Sarracine	CC=C(C)C(=O)OC1CCN2C1C(CC2)COC(=O)C(=CC)CO	5281746	-5.2
77	Matairesinol	COC1=C(C=CC(=C1)CC2COC(=O)C2CC3=CC(=C(C=C3)O)OC)O	119205	-6.2
78	Narciclasine	C1OC2=C(O1)C(=C3C(=C2)C4=CC(C(C(C4NC3=O)O)O)O)O	72376	-5.4
79	Houttuynoid A	CCCCCCCCCC1=C(C2=C(C=CC(=C2O1)O)C3=C(C(=O)C4=C(C=C(C=C4O3)O)O)OC5C(C(C(C(O5)CO)O)O)O)C=O	57412149	-8.2
80	Houttuynoid B	CCCCCCCCCC1=CC2=C(C=CC(=C2O1)O)C3=C(C(=O)C4=C(C=C(C=C4O3)O)O)OC5C(C(C(C(O5)CO)O)O)O	57412150	-6.3
81	Houttuynoid C	CCCCCCCCCC(=O)C1=COC2=C(C=CC(=C12)C3=C(C(=O)C4=C(C=C(C=C4O3)O)O)OC5C(C(C(C(O5)CO)O)O)O)O	57412151	-4.8
82	Houttuynoid D	CCCCCCCCCC(=O)CC1=C(C=CC(=C1O)O)C2=C(C(=O)C3=C(C=C(C=C3O2)O)O)OC4C(C(C(C(O4)CO)O)O)O	57412152	-5.2
83	Houttuynoid E	CCCCCCCCCC(=O)CC1=CC(=C(C=C1C2=C(C(=O)C3=C(C=C(C=C3O2)O)O)OC4C(C(C(C(O4)CO)O)O)O)O)O	57412153	-4.7
84	Hesperidin	CC1C(C(C(C(O1)OCC2C(C(C(C(O2)OC3=CC(=C4C(=O)CC(OC4=C3)C5=CC(=C(C=C5)OC)O)O)O)O)O)O)O)O	10621	-8.6
85	Melittin	CCC(C)C(C(=O)NCC(=O)NC(C)C(=O)NC(C(C)C)C(=O)NC(CC(C)C)C(=O)NC(CCCCN)C(=O)NC(C(C)C)C(=O)NC(CC(C)C)C(=O)NC(C(C)O)C(=O)NC(C(C)O)C(=O)NCC(=O)NC(CC(C)C)C(=O)N1CCCC1C(=O)NC(C)C(=O)NC(CC(C)C)C(=O)NC(C(C)CC)C(=O)NC(CO)C(=O)NC(CC2=CNC3=CC=CC=C32)C(=O)NC(C(C)CC)C(=O)NC(CCCCN)C(=O)NC(CCCNC(=N)N)C(=O)NC(CCCCN)C(=O)NC(CCCNC(=N)N)C(=O)NC(CCC(=O)N)C(=O)NC(CCC(=O)N)C(=O)N)NC(=O)CN	16133648	-6.3
86	Licoisoflavanone	CC1(C=CC2=C(O1)C=CC(=C2O)C3COC4=CC(=CC(=C4C3=O)O)O)C	392443	-4.8
87	Glisoflavanone	CC(=CCC1=C(C=CC(=C1O)C2COC3=C(C2=O)C(=C(C(=C3)O)CC=C(C)C)O)O)C	480786	-6.7
88	Parvisoflavanone	COC1=C(C=CC(=C1OC)O)C2COC3=CC(=CC(=C3C2=O)O)O	44257394	-4.7
89	Sophoraisoflavanone A	CC(=CCC1=C(C=CC(=C1OC)C2COC3=CC(=CC(=C3C2=O)O)O)O)C	442822	-5.6
90	Isohomosovanillic acid	COC1=C(C=C(C=C1)CC(=O)O)O	160562	-7.8

91	Spirooliganone A	CC(C)C12CCC3(C1C2)CCC4=C(O3)C5(CC(C(O5)(C)C)O)C=C(C4=O)CC=C	72946626	-6.7
92	Spirooliganone 3	CC(C)C12CCC3(C1C2)CCC4=C(O3)C5(CC(C(O5)(C)C)O)C=C(C4=O)CC=C	122396353	-6.1
93	Spirooliganone 6	CC(C)C12CCC3(C1C2)CCC4=C(O3)C5(CC(C(O5)(C)C)O)C=C(C4=O)CC=C	122396356	-4.5
94	Spirooliganone 7	CC(C)C12CCC3(C1C2)CCC4=C(O3)C5(CC(C(O5)(C)C)O)C=C(C4=O)CC=C	122396357	-5.0
95	Spirooliganone 8	CC(C)C12CCC3(C1C2)CCC4=C(O3)C5(CC(C(O5)(C)C)O)C=C(C4=O)CC=C	122396358	-6.6
96	Spirooliganone 4	CC(C)C12CCC3(C1C2)CCC4=C(O3)C5(CC(C(O5)(C)C)O)C=C(C4=O)CC=C	122396354	-5.4
97	Spirooliganone 5	CC(C)C12CCC3(C1C2)CCC4=C(O3)C5(CC(C(O5)(C)C)O)C=C(C4=O)CC=C	122396355	-6.1
98	Herbacetin	C1=CC(=CC=C1C2=C(C(=O)C3=C(O2)C(=C(C=C3O)O)O)O)O	5280544	-4.8
99	Myricetin	C1=C(C=C(C(=C1O)O)O)C2=C(C(=O)C3=C(C=C(C=C3O2)O)O)O	5281672	-3.8
100	Formononetin	COC1=CC=C(C=C1)C2=COC3=C(C2=O)C=CC(=C3)O	5280378	-7.2
101	Quercetagetin	C1=CC(=C(C=C1C2=C(C(=O)C3=C(O2)C=C(C(=C3O)O)O)O)O)O	5281680	-7.0
102	Chrysin	C1=CC=C(C=C1)C2=CC(=O)C3=C(C=C(C=C3O2)O)O	5281607	-5.6
103	Sinapic acid	COC1=CC(=CC(=C1O)OC)C=CC(=O)O	637775	-4.5
104	Rhapontigenin	COC1=C(C=C(C=C1)C=CC2=CC(=CC(=C2)O)O)O	5320954	-4.1
105	Pinosylvin	C1=CC=C(C=C1)C=CC2=CC(=CC(=C2)O)O	5280457	-4.6
106	Caffeic acid	C1=CC(=C(C=C1C=CC(=O)O)O)O	689043	-6.7
107	Baicalein	C1=CC=C(C=C1)C2=CC(=O)C3=C(O2)C=C(C(=C3O)O)O	5281605	-6.6
108	Vanillin	COC1=C(C=CC(=C1)C=O)O	1183	-4.6
109	Isoferulic acid	COC1=C(C=C(C=C1)C=CC(=O)O)O	736186	-5.2
110	Irilone	C1OC2=C(O1)C(=C3C(=C2)OC=C(C3=O)C4=CC=C(C=C4)O)O	5281779	-6.7
112	Kaempferol	C1=CC(=CC=C1C2=C(C(=O)C3=C(C=C(C=C3O2)O)O)O)O	5280863	-7.1
113	Coumestrol	C1=CC2=C(C=C1O)OC3=C2C(=O)OC4=C3C=CC(=C4)O	5281707	-7.0
114	Pratensein	COC1=C(C=C(C=C1)C2=COC3=CC(=CC(=C3C2=O)O)O)O	5281803	-6.1
115	Isosakuranetin	COC1=CC=C(C=C1)C2CC(=O)C3=C(C=C(C=C3O2)O)O	160481	-4.1
116	Alpinetin	COC1=CC(=CC2=C1C(=O)CC(O2)C3=CC=CC=C3)O	154279	-5.2
117	Sugiol	CC(C)C1=C(C=C2C(=C1)C(=O)CC3C2(CCCC3(C)C)C)O	94162	-5.3
118	Betulonic acid	CC(=C)C1CCC2(C1C3CCC4C5(CCC(=O)C(C5CCC4(C3(CC2)C)C)(C)C)C)C(=O)O	122844	-6.7
119	Savinin	C1C(C(=CC2=CC3=C(C=C2)OCO3)C(=O)O1)CC4=CC5=C(C=C4)OCO5	5281867	-3.4
120	Xanthoangelol E	CC(=C)C(CC1=C(C=CC(=C1O)C(=O)C=CC2=CC=C(C=C2)O)OC)OO	10022050	-5.4
121	Bavachinin	CC(=CCC1=CC2=C(C=C1OC)OC(CC2=O)C3=CC=C(C=C3)O)C	10337211	-4.5

122	Neobavaisoflavone	CC(=CCC1=C(C=CC(=C1)C2=COC3=C(C2=O)C=CC(=C3)O)O)C	5320053	-5.2
123	Isobavachalcone	CC(=CCC1=C(C=CC(=C1O)C(=O)C=CC2=CC=C(C=C2)O)O)C	5281255	-4.6
124	4’-*O*-methylbavachalcone	CC(=CCC1=CC(=C(C=C1OC)O)C(=O)C=CC2=CC=C(C=C2)OC)C	42607530	-5.2
125	Psoralidin	CC(=CCC1=CC2=C(C=C1O)OC(=O)C3=C2OC4=C3C=CC(=C4)O)C	5281806	-5.4
126	Corylifol	CC(=CCC1=C(C=CC(=C1O)C(=O)C=CC2=CC(=C(C=C2)O)O)O)C	10472405	-6.2
127	Pectolinarin	CC1C(C(C(C(O1)OCC2C(C(C(C(O2)OC3=C(C(=C4C(=C3)OC(=CC4=O)C5=CC=C(C=C5)OC)O)OC)O)O)O)O)O)O	168849	-7.3
128	Rhoifolin	CC1C(C(C(C(O1)OC2C(C(C(OC2OC3=CC(=C4C(=C3)OC(=CC4=O)C5=CC=C(C=C5)O)O)CO)O)O)O)O)O	5282150	-6.1
129	Herbacetin7	C1=CC(=CC=C1C2=C(C(=O)C3=C(O2)C(=C(C=C3O)OC4C(C(C(C(O4)CO)O)O)O)O)O)O	44259932	-6.0
130	Helichrysetin	COC1=CC(=CC(=C1C(=O)C=CC2=CC=C(C=C2)O)O)O	6253344	-4.8
131	Amemtoflavone	C1=CC(=CC=C1C2=CC(=O)C3=C(O2)C(=C(C=C3O)O)C4=C(C=CC(=C4)C5=CC(=O)C6=C(C=C(C=C6O5)O)O)O)O	5281600	-5.6
132	Juglanin	C1=CC(=CC=C1C2=C(C(=O)C3=C(C=C(C=C3O2)O)O)OC4C(C(C(O4)CO)O)O)O	5318717	-3.7
133	Juglanin B	COC1=CC2=CC(=C1O)C3=C(C=CC(=C3)CCC(CCCC2)O)O	44155973	-4.1
134	Glycyrrhizin	CC1(C2CCC3(C(C2(CCC1OC4C(C(C(C(O4)C(=O)O)O)O)OC5C(C(C(C(O5)C(=O)O)O)O)O)C)C(=O)C=C6C3(CCC7(C6CC(CC7)(C)C(=O)O)C)C)C)C	14982	-5.2
135	Indirubin	C1=CC=C2C(=C1)C(=C(N2)O)C3=NC4=CC=CC=C4C3=O	10177	-4.5
136	Indican	C1=CC=C2C(=C1)C(=CN2)OC3C(C(C(C(O3)CO)O)O)O	441564	-3.2
137	Sinigrin	C=CCC(=NOS(=O)(=O)[O-])SC1C(C(C(C(O1)CO)O)O)O.[K+]	23682211	-4.5
138	β-sitosterol	CCC(CCC(C)C1CCC2C1(CCC3C2CC=C4C3(CCC(C4)O)C)C)C(C)C	222284	-6.5
139	Aloeemodin	C1=CC2=C(C(=C1)O)C(=O)C3=C(C2=O)C=C(C=C3O)CO	10207	-3.2
140	Justicidin A	COC1=C(C=C2C(=C1)C(=C3C(=C2OC)COC3=O)C4=CC5=C(C=C4)OCO5)OC	159982	-4.0
141	Tetrandrine	CN1CCC2=CC(=C3C=C2C1CC4=CC=C(C=C4)OC5=C(C=CC(=C5)CC6C7=C(O3)C(=C(C=C7CCN6C)OC)OC)OC)OC	73078	-4.1
142	Fangchinoline	CN1CCC2=CC(=C3C=C2C1CC4=CC=C(C=C4)OC5=C(C=CC(=C5)CC6C7=C(O3)C(=C(C=C7CCN6C)OC)O)OC)OC	73481	-5.2
143	Cepharanthine	CN1CCC2=CC3=C(C4=C2C1CC5=CC=C(C=C5)OC6=C(C=CC(=C6)CC7C8=CC(=C(C=C8CCN7C)OC)O4)OC)OCO3	10206	-6.1
145	Emodin	CC1=CC2=C(C(=C1)O)C(=O)C3=C(C2=O)C=C(C=C3O)O	3220	-5.1
146	Tetra-*O*-galloyl-β-D-glucose	C1=C(C=C(C(=C1O)O)O)C(=O)OCC(C(C(C(C=O)OC(=O)C2=CC(=C(C(=C2)O)O)O)OC(=O)C3=CC(=C(C(=C3)O)O)O)OC(=O)C4=CC(=C(C(=C4)O)O)O)O	101011018	-4.9
147	Tylophorinine	COC1=CC2=C(C=C1)C3=C(CN4CCCC4C3O)C5=CC(=C(C=C52)OC)OC	264751	-6.1
148	Tylophorine N-oxide	COC1=C(C=C2C(=C1)C3=C(C[N+]4(CCCC4C3)[O-])C5=CC(=C(C=C52)OC)OC)OC	10525573	-4.5
149	Tanshinone IIA	CC1=COC2=C1C(=O)C(=O)C3=C2C=CC4=C3CCCC4(C)C	164676	-3.2
150	Tanshinone IIB	CC1=COC2=C1C(=O)C(=O)C3=C2C=CC4=C3CCCC4(C)CO	9926694	-5.4

151	Cryptotanshinone	CC1COC2=C1C(=O)C(=O)C3=C2C=CC4=C3CCCC4(C)C	160254	-4.5
152	Rosmariquinone	CC(C)C1=CC2=C(C3=C(C=C2)C(CCC3)(C)C)C(=O)C1=O	160142	-3.2
153	oleuropein	CC=C1C(C(=COC1OC2C(C(C(C(O2)CO)O)O)O)C(=O)OC)CC(=O)OCCC3=CC(=C(C=C3)O)O	5281544	-5.6
154	Cianidanol	C1C(C(OC2=CC(=CC(=C21)O)O)C3=CC(=C(C=C3)O)O)O	9064	-6.1
155	Kaempferol 3,7-diglucoside	C1=CC(=CC=C1C2=C(C(=O)C3=C(C=C(C=C3O2)OC4C(C(C(C(O4)CO)O)O)O)O)OC5C(C(C(C(O5)CO)O)O)O)O	6325460	-6.7
156	baicalin	C1=CC=C(C=C1)C2=CC(=O)C3=C(C(=C(C=C3O2)OC4C(C(C(C(O4)C(=O)O)O)O)O)O)O	64982	-4.5
157	Nicotianamine	C1CN(C1C(=O)O)CCC(C(=O)O)NCCC(C(=O)O)N	9882882	-4.2
158	Ferruginol	CC(C)C1=C(C=C2C(=C1)CCC3C2(CCCC3(C)C)C)O	442027	-3.9
159	Oroxin B	C1=CC=C(C=C1)C2=CC(=O)C3=C(C(=C(C=C3O2)OC4C(C(C(C(O4)COC5C(C(C(C(O5)CO)O)O)O)O)O)O)O)O	10077207	-5.8
160	Sakuranetin	COC1=CC(=C2C(=O)CC(OC2=C1)C3=CC=C(C=C3)O)O	73571	-6.1
161	Genistein	C1=CC(=CC=C1C2=COC3=CC(=CC(=C3C2=O)O)O)O	5280961	-8.0
162	Genistin	C1=CC(=CC=C1C2=COC3=CC(=CC(=C3C2=O)O)OC4C(C(C(C(O4)CO)O)O)O)O	5281377	-3.1
163	Ipriflavone	CC(C)OC1=CC2=C(C=C1)C(=O)C(=CO2)C3=CC=CC=C3	3747	-5.6
164	Puerarin	C1=CC(=CC=C1C2=COC3=C(C2=O)C=CC(=C3C4C(C(C(C(O4)CO)O)O)O)O)O	5281807	-7.1
167	Glabridin	CC1(C=CC2=C(O1)C=CC3=C2OCC(C3)C4=C(C=C(C=C4)O)O)C	124052	-6.0
168	Diosmin	CC1C(C(C(C(O1)OCC2C(C(C(C(O2)OC3=CC(=C4C(=C3)OC(=CC4=O)C5=CC(=C(C=C5)OC)O)O)O)O)O)O)O)O	5281613	-3.2
169	Diosmetin	COC1=C(C=C(C=C1)C2=CC(=O)C3=C(C=C(C=C3O2)O)O)O	5281612	-6.7
170	Skullcapflavone II	COC1=CC=CC(=C1C2=CC(=O)C3=C(C(=C(C(=C3O2)OC)OC)OC)O)O	124211	-5.6
171	beta-Naphthoflavone	C1=CC=C(C=C1)C2=CC(=O)C3=C(O2)C=CC4=CC=CC=C43	2361	-4.5
172	Orientin	C1=CC(=C(C=C1C2=CC(=O)C3=C(O2)C(=C(C=C3O)O)C4C(C(C(C(O4)CO)O)O)O)O)O	5281675	-3.2
173	Acacetin	COC1=CC=C(C=C1)C2=CC(=O)C3=C(C=C(C=C3O2)O)O	5280442	-4.5
174	Apigenin 7-O-neohesperidoside	CC1C(C(C(C(O1)OC2C(C(C(OC2OC3=CC(=C4C(=C3)OC(=CC4=O)C5=CC=C(C=C5)O)O)CO)O)O)O)O)O	5459217	-7.2
175	Sinensetin	COC1=C(C=C(C=C1)C2=CC(=O)C3=C(C(=C(C=C3O2)OC)OC)OC)OC	145659	-5.6
176	Cirsiliol	COC1=C(C(=C2C(=C1)OC(=CC2=O)C3=CC(=C(C=C3)O)O)O)OC	160237	-3.4
177	Homoplantaginin	COC1=C(C=C2C(=C1O)C(=O)C=C(O2)C3=CC=C(C=C3)O)OC4C(C(C(C(O4)CO)O)O)O	5318083	-5.6
178	Fisetin	C1=CC(=C(C=C1C2=C(C(=O)C3=C(O2)C=C(C=C3)O)O)O)O	5281614	-4.3
179	Quercitrin	CC1C(C(C(C(O1)OC2=C(OC3=CC(=CC(=C3C2=O)O)O)C4=CC(=C(C=C4)O)O)O)O)O	5280459	-5.6
180	Quercetin 3-β-D-glucoside	C1=CC(=C(C=C1C2=C(C(=O)C3=C(C=C(C=C3O2)O)O)OC4C(C(C(C(O4)CO)O)O)O)O)O	5280804	-7.1

181	Kaempferol 7-O-β-D-glucopyranoside	C1=CC(=CC=C1C2=C(C(=O)C3=C(C=C(C=C3O2)OC4C(C(C(C(O4)CO)O)O)O)O)O)O	10095180	-3.2
182	Mangiferin	C1=C2C(=CC(=C1O)O)OC3=C(C2=O)C(=C(C(=C3)O)C4C(C(C(C(O4)CO)O)O)O)O	5281647	-6.2
183	Auraptene	CC(=CCCC(=CCOC1=CC2=C(C=C1)C=CC(=O)O2)C)C	1550607	-4.5
184	Neohesperidin dihydrochalcone	CC1C(C(C(C(O1)OC2C(C(C(OC2OC3=CC(=C(C(=C3)O)C(=O)CCC4=CC(=C(C=C4)OC)O)O)CO)O)O)O)O)O	30231	-7.1
185	Sofalcone	CC(=CCOC1=CC=C(C=C1)C=CC(=O)C2=C(C=C(C=C2)OCC=C(C)C)OCC(=O)O)C	5282219	-6.8
186	Dienestrol	CC=C(C1=CC=C(C=C1)O)C(=CC)C2=CC=C(C=C2)O	667476	-6.6
187	Isoxanthohumol	CC(=CCC1=C2C(=C(C=C1O)OC)C(=O)CC(O2)C3=CC=C(C=C3)O)C	513197	-6.2
188	Astilbin	CC1C(C(C(C(O1)OC2C(OC3=CC(=CC(=C3C2=O)O)O)C4=CC(=C(C=C4)O)O)O)O)O	119258	-4.1
189	Silibinin	COC1=C(C=CC(=C1)C2C(OC3=C(O2)C=C(C=C3)C4C(C(=O)C5=C(C=C(C=C5O4)O)O)O)CO)O	31553	-3.8
190	(+)-*taxifolin*	C1=CC(=C(C=C1C2C(C(=O)C3=C(C=C(C=C3O2)O)O)O)O)O	439533	-6.2
191	Icaritin	CC(=CCC1=C2C(=C(C=C1O)O)C(=O)C(=C(O2)C3=CC=C(C=C3)OC)O)C	5318980	-5.1
192	(−)-Epicatechin	C1C(C(OC2=CC(=CC(=C21)O)O)C3=CC(=C(C=C3)O)O)O	72276	-4.5
193	(±)-Catechin	C1C(C(OC2=CC(=CC(=C21)O)O)C3=CC(=C(C=C3)O)O)OC(=O)C4=CC(=C(C(=C4)O)O)O	5276454	-6.8
194	−) Catechin gallate	C1C(C(OC2=CC(=CC(=C21)O)O)C3=CC(=C(C=C3)O)O)OC(=O)C4=CC(=C(C(=C4)O)O)O	6419835	-7.9
195	(+)-Catechin hydrate	C1C(C(OC2=CC(=CC(=C21)O)O)C3=CC(=C(C=C3)O)O)O.O	107957	-7.0
196	Pelargonidin	C1=CC(=CC=C1C2=[O+]C3=CC(=CC(=C3C=C2O)O)O)O	440832	-4.0
197	Malvidin	COC1=CC(=CC(=C1O)OC)C2=[O+]C3=CC(=CC(=C3C=C2O)O)O	159287	-3.2
198	Pterostilbene	COC1=CC(=CC(=C1)C=CC2=CC=C(C=C2)O)OC	5281727	-4.3
199	Protocatechuic acid	C1=CC(=C(C=C1C(=O)O)O)O	72	-4.5
200	Cinnamic acid	C1=CC=C(C=C1)C=CC(=O)O	444539	-6.1
201	Vanillic acid	COC1=C(C=CC(=C1)C(=O)O)O	8468	-5.6
202	Cis-p-Coumeric acid	C1=CC(=CC=C1C=CC(=O)O)O	1549106	-7.0
203	(+)-epicatechin-3-o-gallate	C1C(C(OC2=CC(=CC(=C21)O)O)C3=CC(=C(C=C3)O)O)OC(=O)C4=CC(=C(C(=C4)O)O)O	65056	-7.9
204	theaflavin	C1C(C(OC2=CC(=CC(=C21)O)O)C3=CC(=O)C(=C4C(=C3)C(=CC(=C4O)O)C5C(CC6=C(C=C(C=C6O5)O)O)O)O)O	4263901	-8.0
205	Theaflavin-3-gallate	C1C(C(OC2=CC(=CC(=C21)O)O)C3=CC4=C(C(=C(C=C4C5C(CC6=C(C=C(C=C6O5)O)O)OC(=O)C7=CC(=C(C(=C7)O)O)O)O)O)O)C(=O)C(=C3)O)O	136825044	-8.2
206	Theaflavin-3,3-digallate	C1C(C(OC2=CC(=CC(=C21)O)O)C3=CC4=C(C(=C(C=C4C5C(CC6=C(C=C(C=C6O5)O)O)OC(=O)C7=CC(=C(C(=C7)O)O)O)O)O)O)C(=O)C(=C3)O)OC(=O)C8=CC(=C(C(=C8)O)O)O	135403795	-7.8
207	Laricitrin	COC1=CC(=CC(=C1O)O)C2=C(C(=O)C3=C(C=C(C=C3O2)O)O)O	5282154	-6.1
208	Isorhamnetin	COC1=C(C=CC(=C1)C2=C(C(=O)C3=C(C=C(C=C3O2)O)O)OC4C(C(C(C(O4)CO)O)O)O)O	5318645	-6.2

209	Resveratrol	C1=CC(=CC=C1C=CC2=CC(=CC(=C2)O)O)O	445154	-6.5
210	Coutaric acid	C1=CC(=CC=C1C=CC(=O)OC(C(C(=O)O)O)C(=O)O)O	57517924	-4.2
211	Dihydrorobinetin	C1=CC2=C(C=C1O)OC(C(C2=O)O)C3=CC(=C(C(=C3)O)O)O	20399	-5.1
212	Robinetin	C1=CC2=C(C=C1O)OC(=C(C2=O)O)C3=CC(=C(C(=C3)O)O)O	5281692	-7.0
213	Tricetin	C1=C(C=C(C(=C1O)O)O)C2=CC(=O)C3=C(C=C(C=C3O2)O)O	5281701	-3.4
214	Norathyriol	C1=C(C=C2C(=C1O)C(=O)C3=CC(=C(C=C3O2)O)O)O	5281656	-4.5
215	Pectolinarigenin	COC1=CC=C(C=C1)C2=CC(=O)C3=C(O2)C=C(C(=C3O)OC)O	5320438	-6.2
216	Urolithin A	C1=CC2=C(C=C1O)C(=O)OC3=C2C=CC(=C3)O	5488186	-7.0
217	Pinocembrin	C1C(OC2=CC(=CC(=C2C1=O)O)O)C3=CC=CC=C3	68071	-5.2
218	Dalbergin	COC1=C(C=C2C(=CC(=O)OC2=C1)C3=CC=CC=C3)O	442768	-4.8
219	Biochanin A	COC1=CC=C(C=C1)C2=COC3=CC(=CC(=C3C2=O)O)O	5280373	-4.6
220	Pinostrobin	COC1=CC(=C2C(=O)CC(OC2=C1)C3=CC=CC=C3)O	73201	-6.2
221	Datiscetin	C1=CC=C(C(=C1)C2=C(C(=O)C3=C(C=C(C=C3O2)O)O)O)O	5281610	-6.3
222	Glycitein	COC1=C(C=C2C(=C1)C(=O)C(=CO2)C3=CC=C(C=C3)O)O	5317750	-5.6
223	Phloretin	C1=CC(=CC=C1CCC(=O)C2=C(C=C(C=C2O)O)O)O	4788	-4.1
224	Angolensin	CC(C1=CC=C(C=C1)OC)C(=O)C2=C(C=C(C=C2)O)O	3584988	-4.4
225	Liquiritigenin	C1C(OC2=C(C1=O)C=CC(=C2)O)C3=CC=C(C=C3)O	114829	-3.1
226	Prunetin	COC1=CC(=C2C(=C1)OC=C(C2=O)C3=CC=C(C=C3)O)O	5281804	-6.0
227	Isorhapontigenin	COC1=C(C=CC(=C1)C=CC2=CC(=CC(=C2)O)O)O	5318650	-6.0
228	Dihydrocaffeic acid	C1=CC(=C(C=C1CCC(=O)O)O)O	348154	-5.9
229	6,7-dehydroroyleanone	CC(C)C1=C(C2=C(C(=O)C1=O)C3(CCCC(C3C=C2)(C)C)C)O	2751794	-4.5
230	Pinusolidic acid	CC12CCCC(C1CCC(=C)C2CCC3=CCOC3=O)(C)C(=O)O	25880646	-5.8
231	Cadinol	CC1=CC2C(CCC(C2CC1)(C)O)C(C)C	6428423	-3.2
232	Hinokinin	C1C(C(C(=O)O1)CC2=CC3=C(C=C2)OCO3)CC4=CC5=C(C=C4)OCO5	442879	-6.9
233	Cryptojaponol	CC(C)C1=C(C(=C2C(=C1)C(=O)CC3C2(CCCC3(C)C)C)O)OC	11724205	-6.1
234	Forskolin	CC(=O)OC1C(C2C(CCC(C2(C3(C1(OC(CC3=O)(C)C=C)C)O)C)O)(C)C)O	47936	-5.0
235	Honokiol	C=CCC1=CC(=C(C=C1)O)C2=CC(=C(C=C2)O)CC=C	72303	-5.1

236	Ginkgolide-B	CC1C(=O)OC2C1(C34C(=O)OC5C3(C2O)C6(C(C5)C(C)(C)C)C(C(=O)OC6O4)O)O	6324617	-5.5
237	Geraniol	CC(=CCCC(=CCO)C)C	637566	-5.2
238	Ginkgolide	CC1C(=O)OC2C1(C34C(=O)OC5C3(C2O)C6(C(C5)C(C)(C)C)C(C(=O)OC6O4)O)O	65243	-5.6
239	Thymol	CC1=CC(=C(C=C1)C(C)C)O	6989	-3.4
240	Steviol	CC12CCCC(C1CCC34C2CCC(C3)(C(=C)C4)O)(C)C(=O)O	452967	-4.8
241	Salvinorin A	CC(=O)OC1CC(C2(CCC3C(=O)OC(CC3(C2C1=O)C)C4=COC=C4)C)C(=O)OC	128563	-5.3
242	Sclareol	CC1(CCCC2(C1CCC(C2CCC(C)(C=C)O)(C)O)C)C	163263	-5.1
243	Manoalide	CC1=C(C(CCC1)(C)C)CCC(=CCCC2=CCC(OC2O)C3=CC(=O)OC3O)C	6437368	-6.0
244	Limonin	CC1(C2CC(=O)C3(C(C24COC(=O)CC4O1)CCC5(C36C(O6)C(=O)OC5C7=COC=C7)C)C)C	179651	-6.1
245	Casimirolide	CC1(C2CC(=O)C3(C(C2(C=CC(=O)O1)C)CCC4(C35C(O5)C(=O)OC4C6=COC=C6)C)C)C	500031	-6.4
246	Paclitaxel	CC1=C2C(C(=O)C3(C(CC4C(C3C(C(C2(C)C)(CC1OC(=O)C(C(C5=CC=CC=C5)NC(=O)C6=CC=CC=C6)O)O)OC(=O)C7=CC=CC=C7)(CO4)OC(=O)C)O)C)OC(=O)C	36314	-4.5
247	Humulone	CC(C)CC(=O)C1=C(C(=C(C(C1=O)(CC=C(C)C)O)O)CC=C(C)C)O	442911	5.4
248	Farnesol	CC(=CCCC(=CCCC(=CCO)C)C)C	445070	5.1

Table S2. Evaluation of Lipinski's rule of 5 with a drug-likeness score by Molsoft L.L.C.: DrugLikeness and molecular property prediction of the selected molecules (Top 4 ligands).

**Compounds**	**Mass (<500)**	**Hydrogen bond donor (<5)**	**Hydrogen bond acceptor (<10)**	**LOGP (<5)**	**Molar Refractivity (40-130)**	**Drug likeness (>0)**
Tellimagrandin I	786.09	22	13	1.01	173.04	0.30
SaikosaponinB2	780.47	9	13	1.68	198.76	0.18
Hesperidin	610.19	8	15	-1.15	140.69	0.94
(-)-Epigallocatechin Gallate	458.08	8	11	2.23	108.92	0.23

Table S3. Metabolism properties of the Tellimagrandin I, SaikosaponinB2, Hesperidin, and (-)-Epigallocatechin Gallate by pKCSM tool.

**Compounds/ Ligands**	**CYP2D6 substrate**	**CYP3A4 substrate**	**CYP1A2 inhibitor**	**CYP2C19 inhibitor**	**CYP2C9 inhibitor**	**CYP2D6 inhibitor**
Tellimagrandin I	No	No	No	No	No	No
SaikosaponinB2	No	No	No	No	No	No
Hespiridin	No	No	No	No	No	No
(-)-Epigallocatechin Gallate	No	No	No	No	No	No

Supplementary Figures

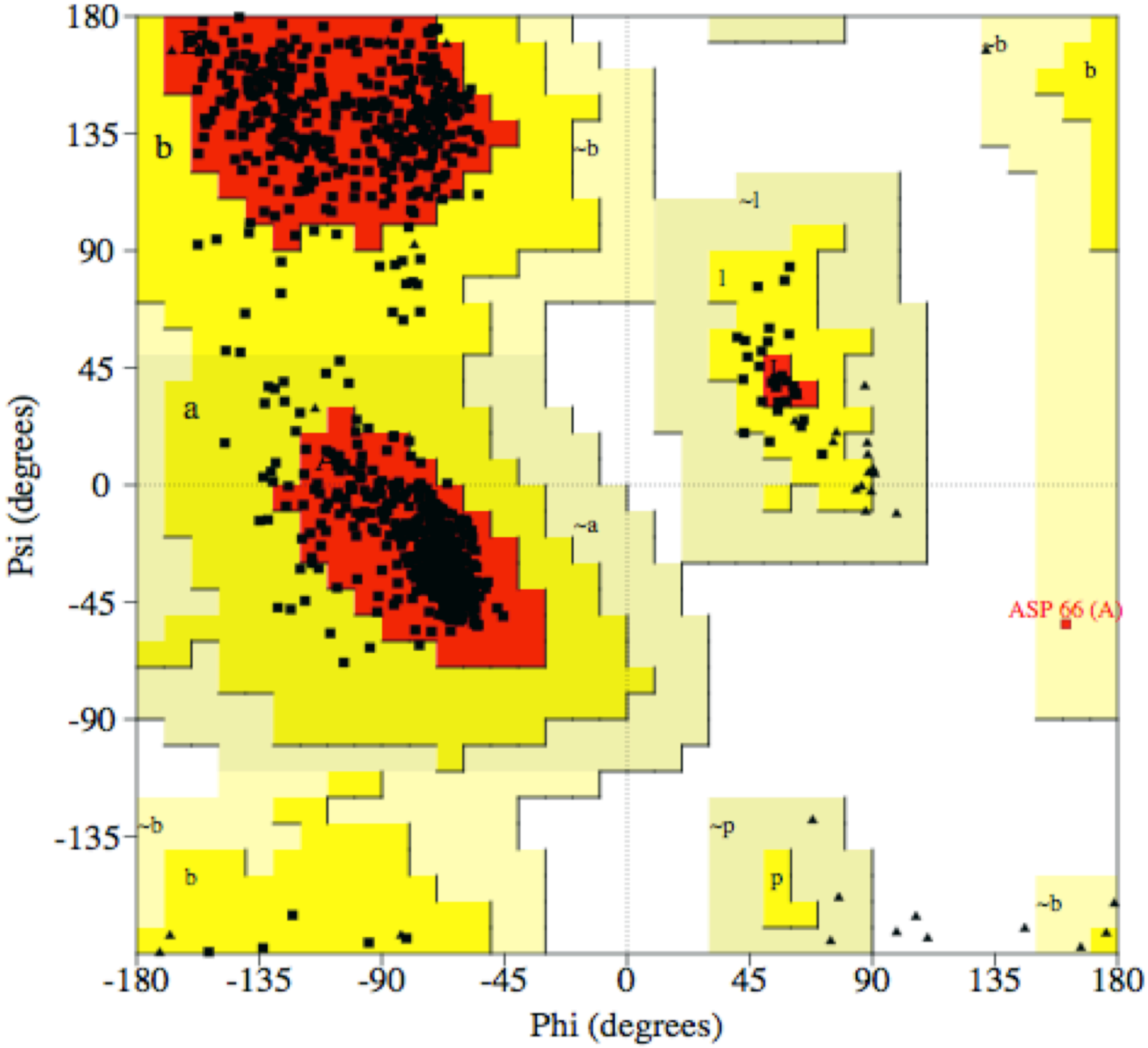

Figure S1. Ramachandran Plot of RNA-dependent RNA polymerase (RdRp) of the SARS-CoV-2.

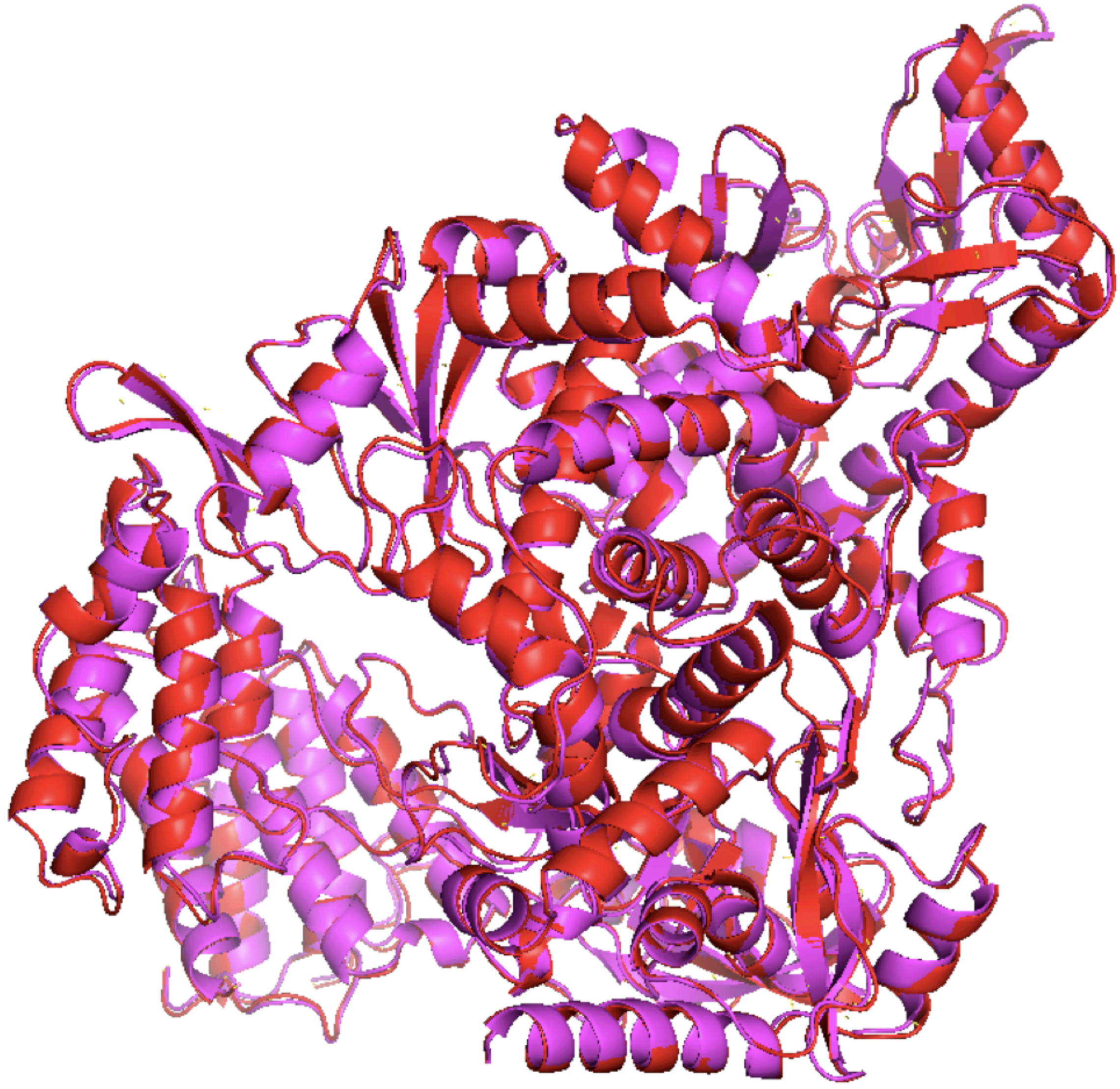

Figure S2. Structure of SARS-CoV-2 RNA-dependent RNA polymerase (RdRp) before (Pink) and after optimization (red), superimposed with RMSD value.

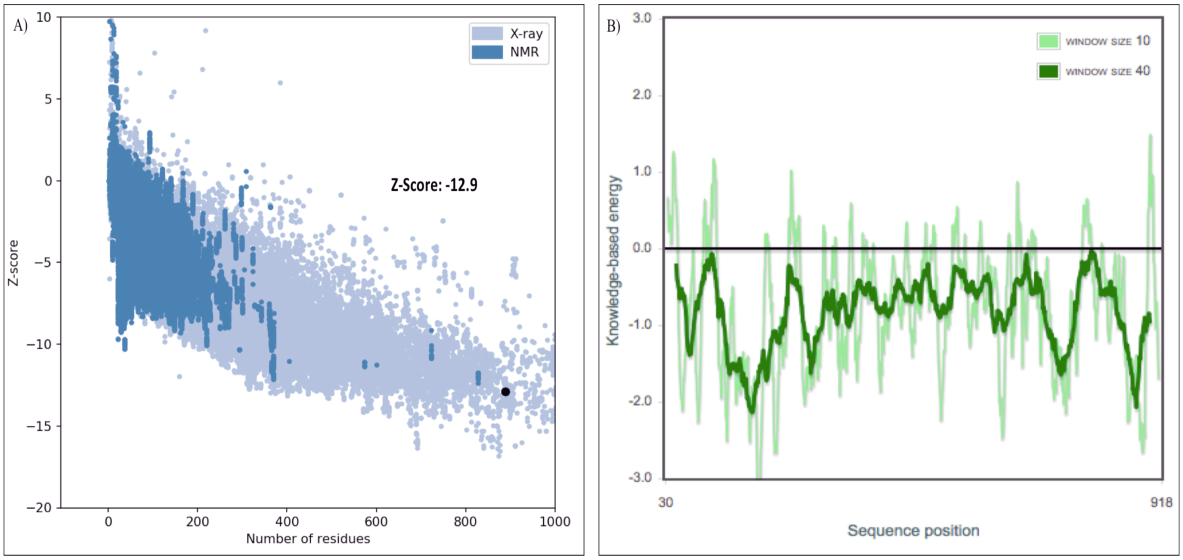

Figure S3. Structure validation of SARS-CoV-2 RdRp by ProSa. (A) Z-scores of all protein chains in PDB as calculated by ProSa that are established by X-ray crystallography (light blue) or NMR spectroscopy (dark blue) concerning protein size. The z-score of RdRp is -12.9 and is underlined with a black dot and **(B)** Energy plot of SARS-CoV-2 RdRp as calculated by ProSA. The thick line indicates average energy with a window size of 40 residues. The thin line indicates average energy over each ten residues fragment in the background of the plot.

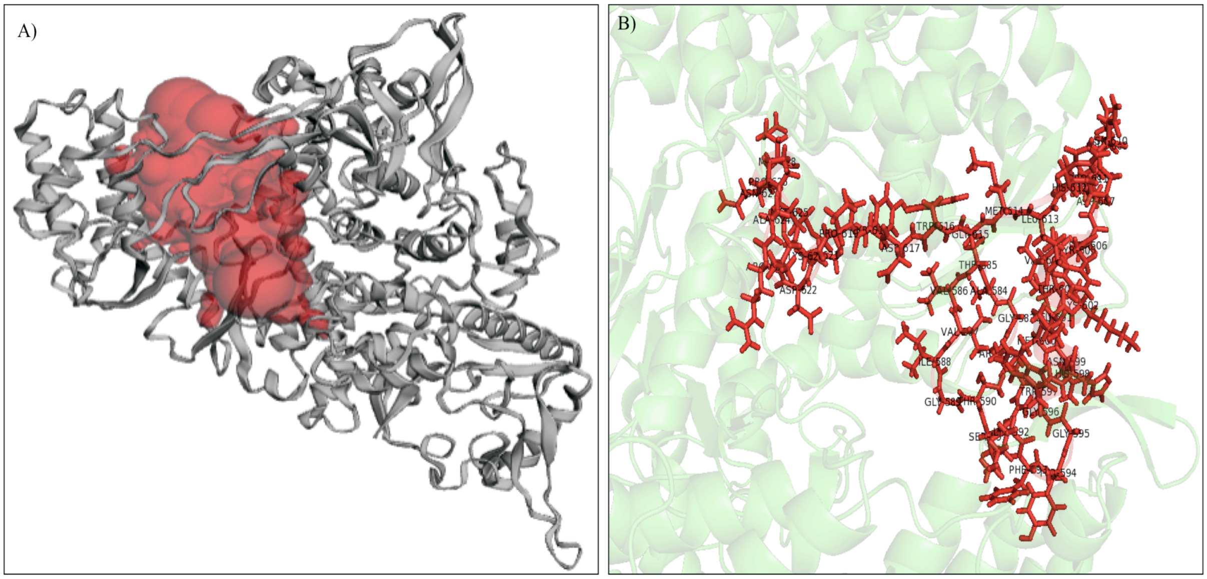

Figure S4. **(A)** Structure of RNA-dependent RNA polymerase (RdRp) of the SARS-CoV-2 with binding pocket (surface and red) as illustrated by CASTp3.0. **(B)** The structure of SARS-CoV-2 RNA-dependent RNA polymerase (RdRp) (cartoon and green) with predicted binding or active sites are highlighted in red.

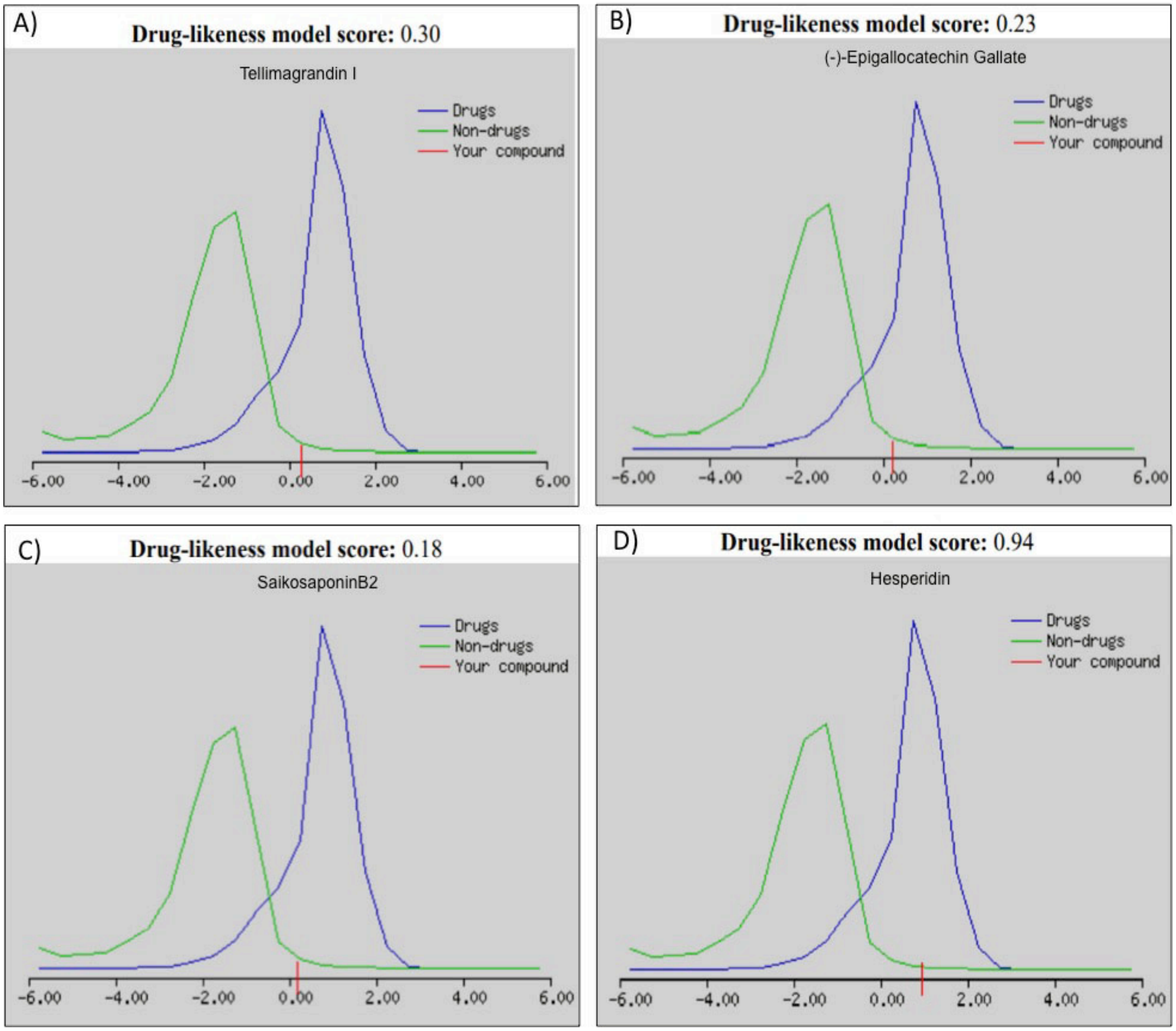

Figure S5. Drug likeness evaluation of selected ligands using Molsoft L.L.C.: Drug-Likeness and molecular property prediction. Tellimagrandin I **(A)**, **(-)-**Epigallocatechin Gallate **(B)**, SaikosaponinB2 **(C)**, and Hesperidin **(D).**
